# *Tetrahymena* ATG8 homologs, TtATG8A and TtATG8B, are responsible for mitochondrial degradation induced by starvation

**DOI:** 10.1128/mbio.00783-25

**Published:** 2025-05-15

**Authors:** Shinya Matsuda, Chieko Saito, Mami Nomura, Hitomi Kawahara, Noboru Mizushima, Kentaro Nakano

**Affiliations:** 1 Degree Programs in Biology, Graduate School of Science and Technology, University of Tsukuba 13121, Tsukuba, Ibaraki Prefecture, Japan; 2 College of Biological Sciences, School of Life and Environmental Sciences, University of Tsukuba 13121, Tsukuba, Ibaraki Prefecture, Japan; 3 Department of Biochemistry and Molecular Biology, Graduate School and Faculty of Medicine, The University of Tokyo 13143, Bunkyo, Tokyo, Japan; 4 Faculty of Science, Yamagata University 13149, Yamagata, Japan; Harvard Medical School, Boston, Massachusetts, USA

**Keywords:** *Tetrahymena*, autophagy, ATG8, mitochondria

## Abstract

The majority of heterotrophic unicellular eukaryotes have evolved mechanisms to survive periods of starvation, allowing them to endure until conditions are favorable for regrowth. The ciliate *Tetrahymena* exhibits active swimming behavior in water, preying on microorganisms and growing exponentially at a rate of 0.5–0.75 h⁻¹ under optimal conditions. In this organism, numerous mitochondria localize to the cell cortex along the ciliary rows, likely ensuring an efficient ATP supply necessary for vigorous cell movement. Although mitochondrial reduction occurs immediately under starvation, the underlying mechanism remains unknown. Here, we demonstrated that autophagy is responsible for mitochondrial reduction in *Tetrahymena thermophila*. Among the five *T. thermophila* ATG8 homologs, TtATG8A and TtATG8B formed granule- and cup-shaped structures in response to starvation. Fluorescent microscopy further showed that TtATG8A and TtATG8B associate with mitochondria. Moreover, correlative light and electron microscopy analysis revealed that mitochondria colocalized with TtATG8A or TtATG8B were engulfed by autophagosomes and displayed abnormal appearances with disrupted cristae structures. Additionally, repression of TtATG8A or TtATG8B expression significantly attenuated starvation-induced mitochondrial reduction. These findings suggest that TtATG8A- and TtATG8B-mediated autophagy is a key mechanism underlying mitochondrial reduction in starved *T. thermophila*.

**IMPORTANCE:**

This study is the first comprehensive description of the mitochondrial degradation process under nutrient starvation in the ciliate *Tetrahymena*. It is well known that the cell surface structure of ciliates consists of an elaborate spatial arrangement of microtubule networks and associated structures and that this surface repetitive pattern is inherited by the next generation of cells like genetic information. Our findings provide a basis for understanding how ciliates maintain an adequate amount of mitochondria on the cell surface in response to nutritional conditions. Furthermore, we have successfully demonstrated the usefulness of *Tetrahymena* as an experimental system for studying mitochondrial quality control and turnover. Further studies of *Tetrahymena* will facilitate comparative studies among diverse biological systems on how eukaryotes other than opisthokonta (yeast, cultured cells, etc.) control their mitochondria.

## INTRODUCTION

Many heterotrophic eukaryotes have evolved strategies to survive periods of starvation, as their nutritional resources are entirely dependent on the extracellular environment. Autophagy is one of the most critical cellular processes triggered by starvation in eukaryotic cells. In organisms that undergo morphological and structural changes in response to their life cycle, defects in autophagy can have severe consequences for these processes (1). Conversely, if vital molecules and organelles are degraded in an unregulated manner, cells face an increased risk of death (2). Therefore, a system ensuring the selective autolysis of specific components is crucial for unicellular eukaryotes to survive in harsh natural environments.

The ciliate *Tetrahymena* is a unicellular eukaryote with highly developed cortical structures, including basal bodies, alveolar sacs, an oral apparatus, and a cytoproct. It is larger than yeast or mammalian cultured cells (3). This organism swims rapidly in water and actively uptakes microorganisms through the oral apparatus, forming a number of phagosomes (food vacuoles) in the cells and then digesting them as a source of nutrients. In *Tetrahymena*, approximately 20 rows of cilia are evenly spaced along the anterior-posterior axis of the teardrop-shaped cell, and their arrangement is believed to be related to the elaborate and regular pattern of cortical cytoskeletal structures, including microtubules. Mitochondria are also localized in the cellular cortex along the ciliary rows, providing ATP necessary for ciliary movement (4). When nutrients are depleted, *Tetrahymena* undergoes conjugation between different mating types, initiating meiosis followed by nuclear exchange. Starvation also triggers notable phenotypic changes in *Tetrahymena*, such as a thinner cell body and increased swimming speed (5, 6). Additionally, in starved cells*,* the number of cortical mitochondria decreases, with remnants of mitochondria found within vacuoles (4, 7, 8). This suggests that *Tetrahymena* cells possess a mechanism to dynamically control mitochondrial numbers to regulate ATP supply for ciliary motility and other cellular activities based on their nutritional status. However, the molecular mechanism behind this regulation remains unclear.

Autophagy, a self-degradation system for cellular components, is one strategy cells use to eliminate unnecessary intracellular components (9). Autophagy can be broadly categorized into three types: macroautophagy, microautophagy, and chaperone-mediated autophagy. Among these, macroautophagy has been extensively studied across various species and will be referred to as autophagy for simplicity hereafter. The basic principle of autophagy is as follows: membrane cisterna, known as an isolation membrane or phagophore, which engulfs cytoplasmic components to form autophagosomes. These autophagosomes then fuse with lysosomes (in mammals) or vacuoles (in yeast and plants), where the engulfed substances are hydrolyzed. The formation of autophagosomes is regulated by several protein complexes composed of “core ATG proteins,” including the ATG1/ULK1 complex, ATG9 vesicles, PI3K complex, ATG2-ATG18 complex, and ATG8-phosphatidylethanolamine (PE) conjugation system. Among them, ATG8-PE, a product of the ATG8-PE conjugation system, has been well studied as a marker for detecting autophagy since it is abundantly localized to autophagosomes and their precursors (10). ATG8 is a ubiquitin-like protein that is cleaved on the C-terminal side of the molecule by the cysteine protease ATG4 to expose its glycine residue, where the E1-like enzyme ATG7 and the E2-like enzyme ATG3 function in concert to finally add PE (11). ATG8-PE induces the formation of the autophagosome, together with the ATG12-ATG5-ATG16 complex, the product of another ubiquitin-like pathway that is characteristic of autophagy (12). Thus, ATG8 is directly associated with membranes to promote autophagosome formation. In addition, ATG8 also plays a crucial role in the selective recognition of degradation targets (13). A portion of ATG8-PE is proteolyzed during autophagosome degradation by lysosomal enzymes, but the remaining is recycled (11).

In *Tetrahymena thermophila*, two ATG8 homologs, TtATG8A (ATG8-65) and TtATG8B (ATG8-2), were first identified by Liu and Yao (14) and were later found to be part of a total of five homologs (15). While the role of TtATG8s immediately after starvation remains unclear, TtATG8A and TtATG8B are involved in the degradation of the parental macronucleus during mating (14). Given that excess mitochondria are removed via autophagy in various organisms (16), it is possible that mitochondrial reduction in starved *Tetrahymena* is also mediated by autophagy. In this study, we investigated the relationship between TtATG8s and mitochondrial degradation during starvation. Based on subcellular localization patterns and gene repression phenotypes, TtATG8A and TtATG8B were found to play a crucial role in starvation-induced mitochondrial degradation. Correlative light and electron microscopy (CLEM) analysis further confirmed that TtATG8A and TtATG8B are involved in both autophagosome formation and mitochondrial degradation. This study offers valuable insights into the mechanisms regulating mitochondrial numbers in *Tetrahymena* and broadens our knowledge of the physiological functions of autophagy in ciliates.

## RESULTS

### Nutrient depletion induces mitochondrial degradation in *T. thermophila*

**Fig 1:**
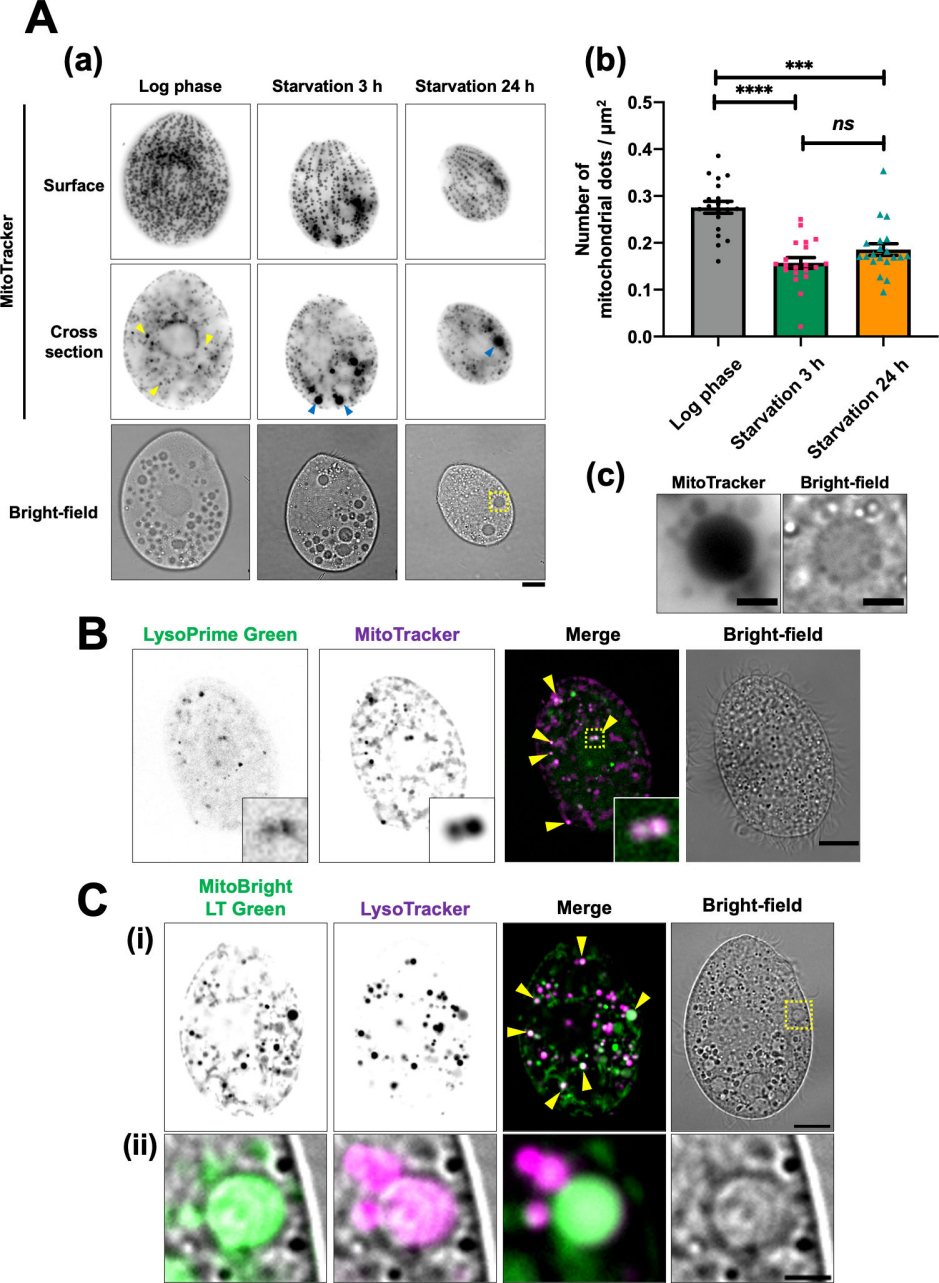
Cortical mitochondria are reduced in starved cells, likely due to their association with digestive organelles such as lysosomes and vacuoles. (**A**) *T. thermophila* wild-type cells were cultured in super proteose peptone medium until they reached log phase and then transferred to 10 mM Tris-HCl (pH 7.5) for starvation. The cells were stained with 200 nM MitoTracker Red CMXRos for at least 30 min. Fluorescent images were captured using an Olympus BX51 microscope. Due to the frequent movement of vacuoles within living cells, the fluorescent and bright-field images may not always align precisely. Scale bars: 10 µm (a). The number of mitochondrial dots and cell area (µm^2^) were quantified using Image J software. Results are presented as the mean ± s.e.m (*n* = 20 cells). One-way analysis of variance followed by Tukey’s multiple comparison test was used, with ***P < 0.001, ****P < 0.0001, and *ns* indicating not significant (b). (c) A magnified view of the yellow dotted square in panel a. Scale bar: 3 µm. (**B**) Two-hour starved wild-type cells were stained with LysoPrime Green (green) and MitoTracker Red CMXRos (magenta) for 1 h. The stained cells were fixed with 2% paraformaldehyde, and fluorescence images were obtained using a Thunder imaging system. Yellow arrowheads indicate colocalization of mitochondria and lysosomes. The bottom right inset shows a magnified view of the yellow dotted square. (**C**) After 2 h of starvation, wild-type cells were simultaneously stained with MitoBright LT Green (green) and LysoTracker Red DND-99 (magenta) for at least 30 min. Fluorescent images were captured using a Thunder imaging system. Yellow arrowheads indicate colocalization of mitochondria and LysoTracker. The bottom panel (ii) shows a magnified view of the yellow dotted square in the upper panel (i). Each fluorescence image is merged with a bright-field image. Scale bars: 10 µm (i) and 2 µm (ii).

To assess mitochondrial reduction following nutrient depletion, we first stained *T. thermophila* with MitoTracker, a fluorescent probe that selectively permeates and accumulates inside mitochondria (17). MitoTracker-stained living cells were held in place with a cover glass to clearly visualize the cell surface, though this caused slight flattening of the cells (details are described in Materials and Methods). As previously reported (4, 18), most mitochondria were aligned along the cellular cortex, with a few cytoplasmic mitochondria (yellow arrowhead) scattered throughout the cell interior during the log phase (Fig. 1A-a). After 3 h of starvation, the density of cortical mitochondria was significantly reduced (surface in Fig. 1A-a and b), and large MitoTracker-stained dots (blue arrowhead) were frequently observed in the cytoplasm (cross-section in Fig. 1A-a). Those dots closely resembled vacuoles in shape, size, and localization (Fig. 1A-c), suggesting that some mitochondria accumulated in vacuoles during nutrient depletion. Previous studies have shown that cell size gradually decreases with prolonged starvation (5, 6). On the other hand, there was no significant difference in mitochondrial density after 24 h of starvation compared to that observed at 3 h (Fig. 1A-b). These results indicate that cortical mitochondria are rapidly reduced in response to starvation and maintain a low density during prolonged starvation as the cells become smaller.

Previous studies have identified two types of lysosomes in *Tetrahymena* cells: primary lysosomes, which are small and have high electron density, and secondary lysosomes, which are formed when the primary lysosomes fuse with vacuoles such as phagosomes (19, 20). Degraded mitochondria have been observed in vacuoles in starved *T. pyriformis* (7, 8). We investigated the relationship between mitochondria and digestive organelles, such as lysosomes and vacuoles, in starved *T. thermophila*. First, we stained cells with LysoPrime Green, a fluorescent probe for lysosomes, to examine whether mitochondria are incorporated in lysosomes. As shown in Fig. 1B, the staining revealed multiple small dots (less than 1 µm in diameter) scattered throughout the cytoplasm, many of which were primary lysosomes based on their size. Since vacuoles were scarcely observed in LysoPrime Green-stained cells, this probe appears to be highly selective for lysosomes. Importantly, these lysosomes frequently colocalized with cytoplasmic mitochondria in starved cells (see inset in Fig. 1B). We further assessed the acidity of lysosomes using the lysosome-directed pH indicator pHLys Red, which fluoresces at pH levels below 5.5. As shown in Fig. S1A (yellow arrowheads), lysosomes were observed as small puncta in the cytoplasm, some of which colocalized with pHLys Red in *T. thermophila* cells. Additionally, LysoTracker, a probe for acidic organelles, fluoresced in some vacuoles, indicating that these fluorescing vacuoles were likely secondary lysosomes (Fig. 1C-ii). Notably, LysoTracker-labeled vacuoles frequently colocalized with large mitochondrial puncta (Fig. 1C-ii). Furthermore, in cells in which FITC-dextran was incorporated into the phagosomes by phagocytosis, large mitochondria puncta were found to localize in the fluorescently labeled phagosomes (yellow arrowheads in Fig. S1B). Since the mitochondria within cells are not typically phagocytosed, this finding suggests that the mitochondria wrapped in autophagosomes may have fused with the phagosomes. Thus, phagosomes may contribute to the bulk degradation of mitochondria wrapped in autophagosomes, as described in the next section. Given that autophagy mediates the transport of mitochondria to lysosomes in animal cells and vacuoles in yeast (16), it is likely that autophagy induces mitochondrial degradation in autolysosomes and phagolysosomes in starved *T. thermophila*.

### Subcellular localization of *Tetrahymena* ATG8 homologs

We investigated whether ATG8 was involved in mitochondrial degradation in *T. thermophila*, given its role as a reliable marker of autophagy and its central role in this pathway (10). *T. thermophila* contains five ATG8 homologs: *TtATG8A*/*ATG8-65* (TTHERM_00522490), *TtATG8B*/*ATG8-2* (TTHERM_00037460), *TtATG8C* (TTHERM_000780499), *TtATG8D*/*ATG8-66* (TTHERM_00526360), and *TtATG8F* (TTHERM_00500940) (14, 15). TtATG8A and TtATG8B possess an ATG4 target site with a C-terminal glycine residue, similar to typical ATG8 proteins found in other organisms, including the budding yeast *Saccharomyces cerevisiae*. Recent studies have suggested that TtATG8B may be modified by PE during programmed nuclear degradation in sexual reproduction (21). In contrast, TtATG8C retains an exposed C-terminal glycine residue, while TtATG8F lacks this residue. Additionally, TtATG8D contains a transmembrane domain instead of a C-terminal glycine, indicating that TtATG8D may be constitutively associated with membranes (15).

**Fig 2:**
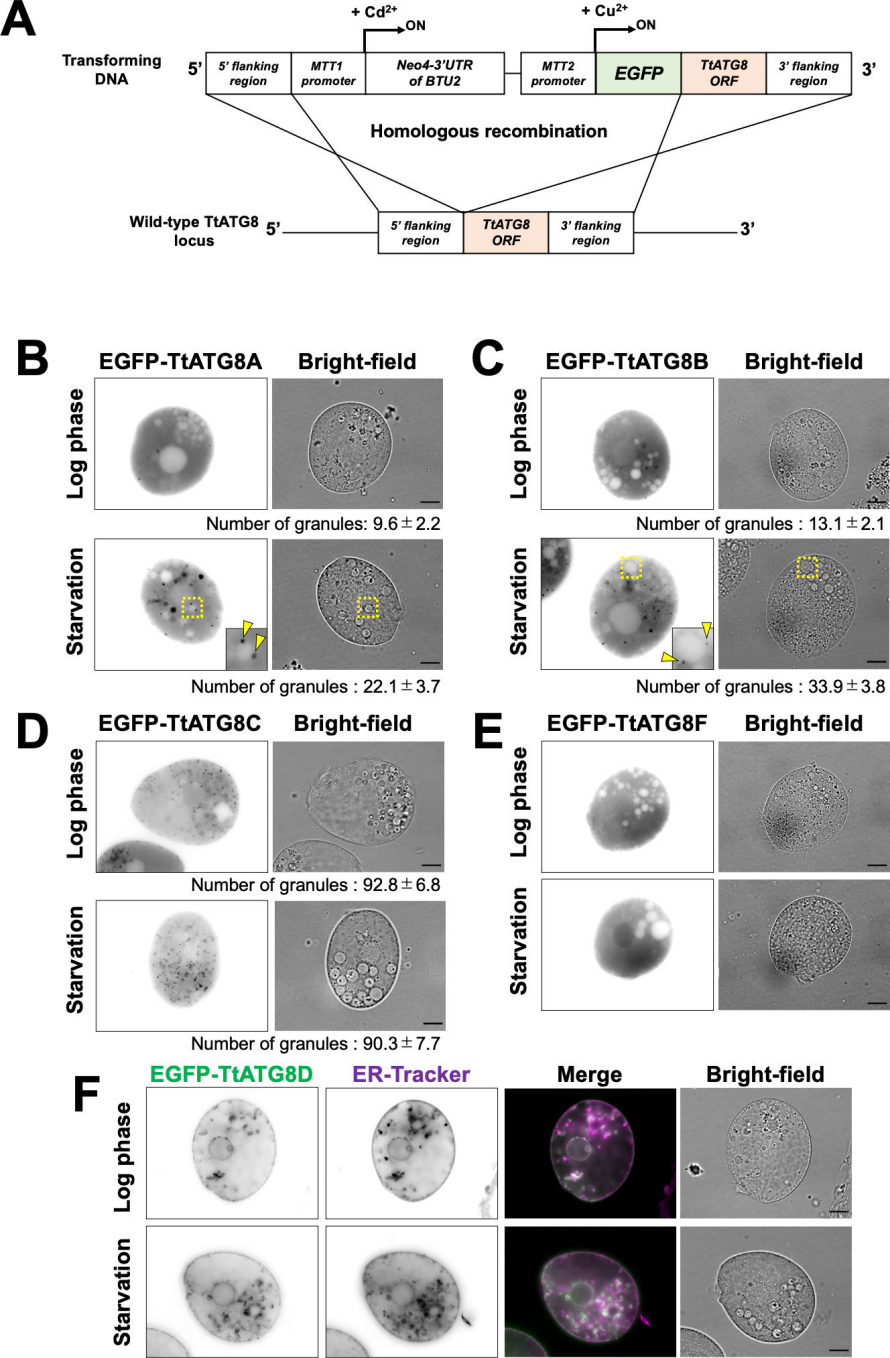
Localization pattern of EGFP-TtATG8s during starvation. (**A**) Illustration of homologous recombination at the *TtATG8s* gene locus. (**B through F**) Cells expressing the indicated *EGFP-TtATG8* constructs were cultured in Super Proteose Peptone medium containing 1.5 mM CuSO_4_ for 4–8 h. Log phase or 3 h-starved cells were observed using an Olympus BX51 microscope. The fluorescence images were focused on cross-sections of the cells. The number of EGFP-TtATG8 granules is shown below each panel. Results are presented as the mean ± s.e.m (*n* = 10 cells). Statistical significance was determined using Welch’s *t*-test (unequal variance assumed). *P*-values: log phase vs starvation in panel B, *P* = 0.0107; log phase vs starvation in panel C, *P* = 0.0003. In panel F, cells were stained with ER-Tracker 1 h prior to observation. Green indicates EGFP-TtATG8D, and magenta indicates ER-Tracker. Scale bars: 10 µm.

To investigate the subcellular localization of TtATG8s, we substituted each *TtATG8* locus with a cassette containing a Cu^2+^-inducible promoter *MTT2* (22) and the coding sequence of the corresponding protein, N-terminally fused to EGFP, via homologous recombination (Fig. 2A). We assessed MTT2-driven *TtATG8* expression levels by quantitative PCR (see supplemental Materials and Methods and Fig. S2 and S3). *TtATG8B* showed no significant difference between MTT2-driven expression and endogenous levels after 3 h of starvation (Fig. S3B). In addition, *TtATG8A* expression under MTT2 was about only threefold higher than endogenous levels (Fig. S3A). To the best of our knowledge, no case of mis-localization is known for overexpressed ATG8. The threefold increase in expression may not have a serious influence on this experiment. In cells treated with Cu^2+^, EGFP-TtATG8A and EGFP-TtATG8B were uniformly localized in the cytoplasm, forming a small number of cytoplasmic granules during the log phase (Fig. 2B and C). After 3 h of starvation, the number of granules increased two- to threefold. Notably, the granules appeared to enlarge and were often observed in contact with structures resembling vacuoles, characterized by circular areas with a thin cytoplasmic background (yellow arrowheads in Fig. 2B and C). In contrast, TtATG8C also formed granules, but there was no significant change in the number of granules before and after starvation (Fig. 2D). TtATG8F localized to both the cytoplasm and nucleus without forming granules during both the log phase and starvation (Fig. 2E). Additionally, TtATG8D appeared to be localized to the ER, with no alteration in localization pattern upon starvation (Fig. 2F). Interestingly, according to the TetraFGD database (http://tfgd.ihb.ac.cn), the transcription levels of *TtATG8A* and *TtATG8B,* but not *TtATG8D* and *TtATG8F,* increased immediately after starvation (Fig. S4), while no data were available for TtATG8C. Given these localization patterns and transcription responses, we focused on the roles of TtATG8A and TtATG8B in mitochondrial degradation for further investigation.

### Association of TtATG8A and TtATG8B with mitochondria

**Fig 3:**
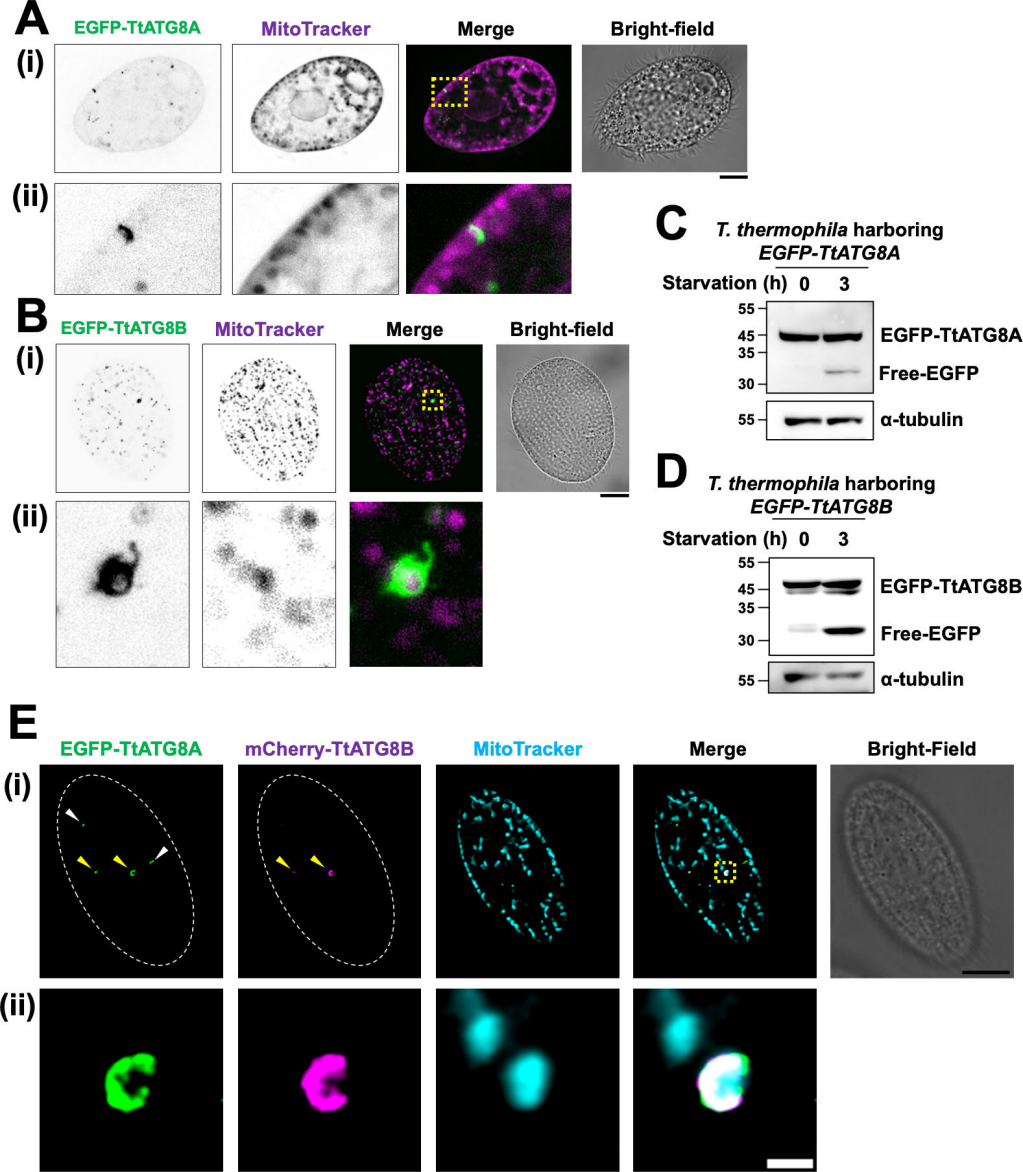
*TtATG8A* and *TtATG8B* in contact with mitochondria. (A–D) *T. thermophila* strains expressing *EGFP-TtATG8A* or *EGFP-TtATG8B* strains were grown in Super Proteose Peptone with 1.5 mM CuSO_4_, starved for 2 h, and then stained with 200 nM MitoTracker Red CMXRos for 1 h. The cells were fixed with 2% paraformaldehyde, and fluorescence images were captured using deconvolution microscopy with the Thunder Imaging System. The bottom panel (ii) provides a magnified view of the yellow dotted square from the upper panel (i). Green: EGFP-TtATG8A; magenta: MitoTracker. Scale bar: 10 µm (**A and B**). The fluorescence images were focused on cross-sections of the cells (**A**) or cell surface (**B**), respectively. Western blot analysis of cell lysates using anti-GFP and anti-α-tubulin antibodies is shown in panels C and D. α-tubulin was used as a protein loading control. (**E**) *T. thermophila* strains expressing *EGFP-TtATG8A* and *mCherry-TtATG8B* strains were cultured in super proteose peptone with 1.5 mM CuSO_4_, starved for 2 h, and then stained with 200 nM MitoTracker DeepRed for 1 h. The cells were fixed with 2% paraformaldehyde, and fluorescence images were obtained using a TCS SP8 confocal microscope. Green: EGFP-TtATG8A; magenta: mCherry-TtATG8B, and cyan: MitoTracker. The bottom panel (ii) shows a magnified view of the yellow dotted square from upper panel (i). Scale bars: 10 µm (i) and 1 µm (ii).

We next examined whether TtATG8A and TtATG8B interact with mitochondria. As a result, it was frequently observed that their co-localization occurred both near the cell surface and within the cytoplasm after induction of starvation (Fig. S5 to S8; see below). Moreover, we performed a detailed study of their co-localization using deconvolution microscopy (Fig. 3A and B). This technique helps to clearly detect protein-mitochondria interactions by minimizing fluorescence signal leakage from outside the focal plane, an important consideration given the large size and thick cytoplasm of *Tetrahymena*. In cells starved for 3 h, EGFP-TtATG8A appeared as small dots or disk-shaped structures in contact with cortical mitochondria (Fig. 3A-ii). Similarly, EGFP-TtATG8B formed vesicular structures that engulfed mitochondria in a region near the cell surface (Fig. 3B-ii). These observations indicate that TtATG8A and TtATG8B interact with mitochondria during starvation, with interactions occurring near the cell surface. In addition, granular localization of TtATG8A and TtATG8B, which were not in contact with mitochondria, was also observed, but it could not be determined whether they were about to contact mitochondria or were involved in autophagy of non-mitochondrial materials from the observation this time.

Free GFP, a degradation product of GFP-ATG8, is commonly used to assess autophagy levels (23). Additionally, conjugation of the C-terminal of ATG8 with PE is important for its binding to the membrane in the progression of autophagy. Urea-SDS-PAGE has been used to quantify the ratio of non-lipidated ATG8 to ATG8-PE in various organisms (24–26). We, therefore, evaluated whether TtATG8A and TtATG8B were conjugated to PE and degraded after starvation by immunoblot analysis. As shown in Fig. 3C and D, free EGFP was detected at 30–35 kDa after 3 h of starvation in both EGFP-TtATG8A- and EGFP-TtATG8B-expressing cells, indicating that both TtATG8A and TtATG8B were proteolytically digested in autolysosomes. Furthermore, it was found that an increase in a small band of TtATG8B, which might be the PE-conjugation form, was observed in response to starvation, although only one band corresponding to EGFP-TtATG8A was observed (Fig. 3C and D). It is possible that TtATG8A and TtATG8B may have different PE-conjugation efficiencies or different turnover rates of conjugation and deconjugation with PE. Indeed, it has been demonstrated that PfATG8 is detected in extracts from *Plasmodium falciparum* cells as a single band because almost all of the protein is of the PE-conjugation form (27). However, it cannot be excluded that EGFP-TtATG8A-PE and the non-lipidated form may not have been separated clearly under the conditions of our electrophoresis.

### TtATG8A and TtATG8B function independently in mitochondrial interaction

To compare the localization patterns of TtATG8A and TtATG8B, we constructed cells expressing both EGFP-TtATG8A and mCherry-TtATG8B (Fig. 3; Fig. S9). TtATG8A granules were observed both with (indicated by yellow arrowheads) and without (white arrowheads) colocalization with TtATG8B (Fig. 3E-i). Similarly, TtATG8B granules without TtATG8A colocalization were also detected (Fig. S9A, white arrowheads). These observations suggest that the formation of TtATG8A and TtATG8B granules can occur independently. However, when these granules grew larger near mitochondria or formed cup-like structures, colocalization of both proteins was frequently observed (see Fig. 3E-ii; Fig. S9A and B, yellow arrowheads), although this was not always the case (a white arrowhead in Fig. S9B-ii). These results indicate that while TtATG8A and TtATG8B can function independently in their interaction with mitochondria, they often co-localize when forming larger structures around mitochondria. Thus, TtATG8A and TtATG8B are not mutually exclusive in their roles on mitochondrial degradation and may collaborate in forming the cup structures that engulf the mitochondria.

### TtATG8A and TtATG8B mediate autophagic degradation of mitochondria

**Fig 4:**
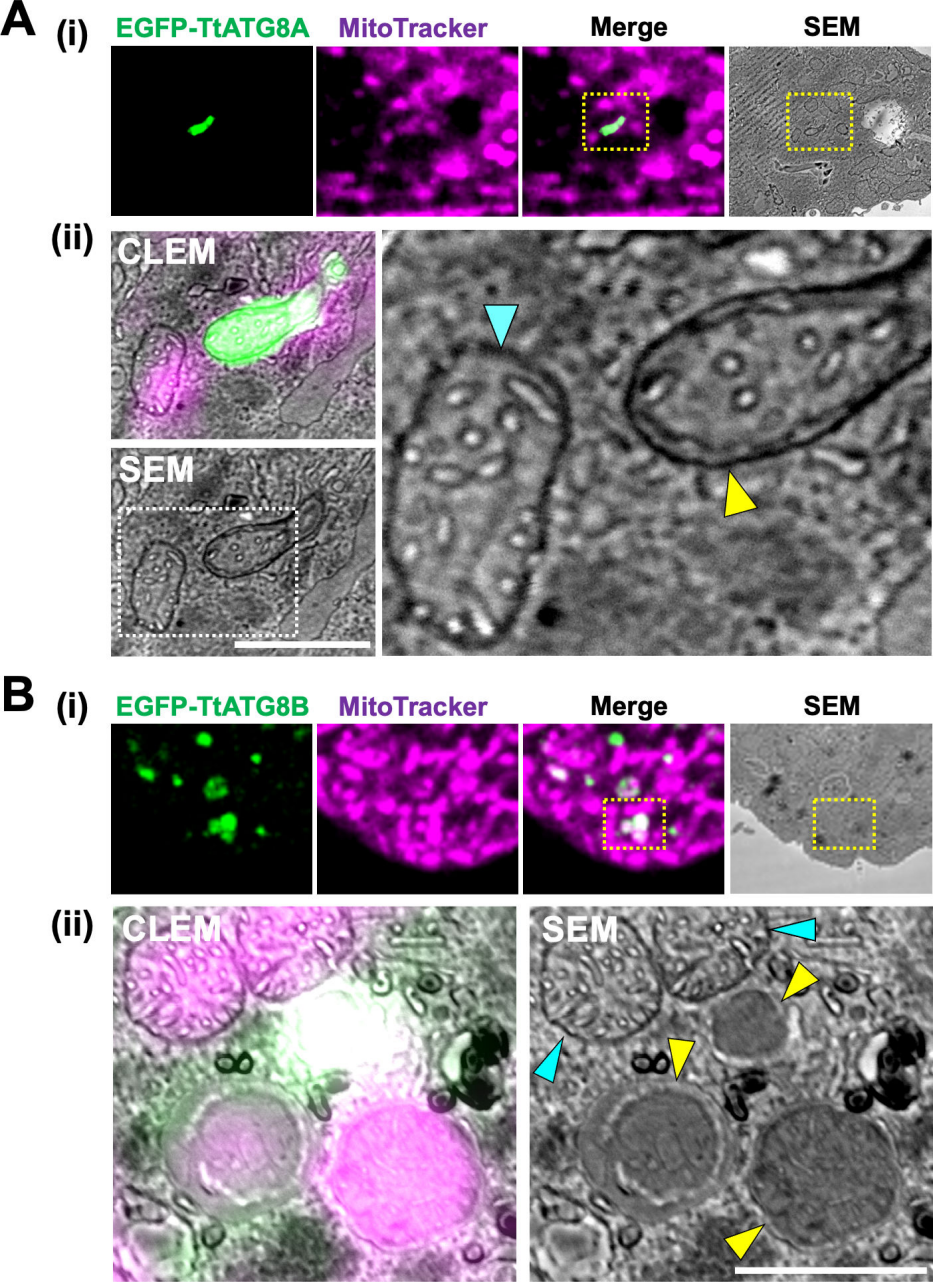
CLEM analysis of TtATG8A and TtATG8B. (**A and B**) Fluorescence, SEM, and CLEM images of *T. thermophila* expressing EGFP-TtATG8A (**A**) and EGFP-TtATG8B (**B**) after 3 h of starvation. The bottom panel (ii) shows a magnified view of the yellow dotted square from the upper panel (i). (A-ii) The right panel provides a magnified view of the white dotted square from the SEM image. Blue arrowheads indicate the outer membrane of mitochondria. Yellow arrowheads indicate autophagosome-like membranes. Scale bars: 1 µm.

Fluorescence microscopy indicated that mitochondrial elimination might be mediated by TtATG8A and TtATG8B (Fig. 3), but it was unclear whether mitochondria associated with these proteins are engulfed in autophagosomes and degraded at the ultrastructural level. To address this, we performed CLEM analysis to investigate the ultrastructure of mitochondria interacting with TtATG8A or TtATG8B. First, we analyzed the spatial distribution of TtATG8A and TtATG8B by fixing 3 h-starved cells expressing each fluorescent protein. Serial sections of these cells were scanned with confocal laser microscopy, and 3D reconstructions were generated. We observed that TtATG8A formed elongated structures adjacent to mitochondria near the cell surface (yellow arrowhead in Fig. S10A). Similarly, TtATG8B was found to contact mitochondria, with some instances forming granular structures that overlapped with the mitochondria (yellow arrowheads in Fig. S10B-ii). According to the 3D reconstructions, 20% of TtATG8A and 24% of TtATG8B granules were in contact with or very close to mitochondria (Fig. S10C). Subsequently, serial thin sections of these cells were prepared for scanning electron microscopy (SEM). The images were merged to produce CLEM images. As shown in Fig. 4A-i, and B-i, mitochondria engulfed by a membrane structure, presumably an autophagosome, were observed in areas indicated by yellow dotted boxes. These areas were then re-scanned at high magnification (Fig. 4A-ii and B-ii). Mitochondria labeled with EGFP-TtATG8A were surrounded by an electron-dense membrane, which is presumed to be a double membrane (yellow arrowheads in Fig. 4A-ii and Fig. S11A-ii). In addition, mitochondria labeled with EGFP-TtATG8B were encased in double membrane structures resembling autophagosomes (yellow arrowheads in Fig. 4B-ii and Fig. S11B-ii), unlike intact mitochondria (blue arrowheads in Fig. 4A-ii and B-ii). Furthermore, the outer membrane of mitochondria co-localizing with EGFP-TtATG8B was unclear, and the cristae structures were only partially visible (Fig. 4B-ii), suggesting that these mitochondria might be undergoing digestion. In summary, CLEM analysis demonstrated that mitochondria colocalized with TtATG8A or TtATG8B were engulfed by autophagosomes and eventually degraded during starvation.

### TtATG8A and TtATG8B are required for mitochondrial degradation

**Fig 5:**
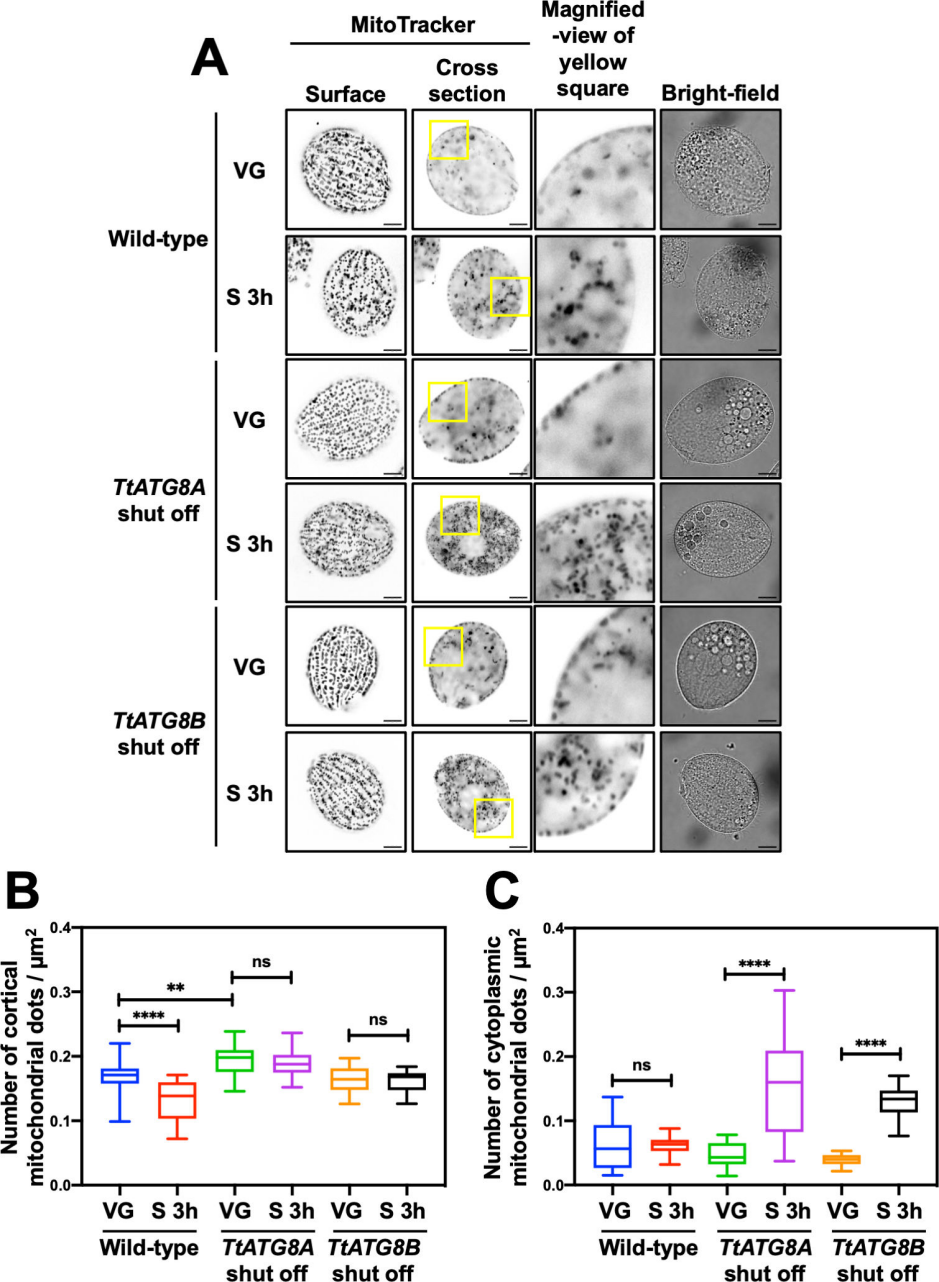
Shutoff of *TtATG8A* and *TtATG8B* suppresses mitochondrial degradation after starvation. (**A**) *T. thermophila* cells, where *TtATG8A* or *TtATG8B* genes on the macronuclear chromosomes were almost completely replaced with the corresponding EGFP-TtATG8 cassette (see Fig. 2A), were cultured in Super Proteose Peptone without CuSO_4_. Vegetative growth cells (VG) or 3 h-starved cells (S 3 h) were stained with 200 nM MitoTracker Red CMXRos. Fluorescent images were captured using a Thunder imaging system with deconvolution fluorescence microscopy. Scale bars: 10 µm. (**B and C**) The number of cortical or cytoplasmic mitochondrial dots and cell area (µm^2^) were quantified using Image J software. Results are presented as box-and-whisker plots showing the median, interquartile range, and minimum/maximum values (*n* = 20 cells). One-way analysis of variance followed by Tukey’s multiple comparison test was used, with **P < 0.01, ****P < 0.0001, and ns indicating not significant.

To assess the roles of TtATG8A and TtATG8B in mitochondrial degradation, we used *T. thermophila* cells with the original *TtATG8A* or *TtATG8B* loci replaced by *MTT2-EGFP-TtATG8A* or *MTT2-EGFP-TtATG8B*, respectively. These cells were grown without Cu^2+^ to repress the expression of each *TtATG8* gene individually. Quantitative PCR analysis also confirmed that the expression levels of *TtATG8A* and *TtATG8B* were significantly reduced in each *TtATG8* shutoff strain compared to those in wild-type cells (Fig. S3). When EGFP-TtATG8A or EGFP-TtATG8B was expressed under Cu^2+^ induction, mitochondrial density decreased under starvation conditions to levels comparable to those in wild-type cells (Fig. S12A and B). We then quantified mitochondrial density in the cellular cortex and cytoplasm using MitoTracker staining and deconvolution fluorescence microscopy (Fig. 5A). In wild-type cells, mitochondrial density significantly decreased after 3 h of starvation. However, in cells where *TtATG8A* or *TtATG8B* expression was repressed, there was no significant reduction in mitochondrial density either before and after starvation (Fig. 5A and B). These results suggest that both TtATG8A and TtATG8B are necessary for the degradation of mitochondria in response to starvation. Interestingly, during vegetative growth, mitochondrial density in *TtATG8A* shut-off cells was slightly higher compared to that in wild-type and *TtATG8B* shut-off cells (Fig. 5B). This suggests that *TtATG8A* might play a role in maintaining mitochondrial quality through homeostatic autophagy, even under nutrient-rich conditions. Furthermore, under starvation conditions, mitochondria that have detached from the cortical region are more prominently retained in the cytoplasm in *TtATG8A* or *TtATG8B* shut-off strains compared to the wild type (Fig. 5A and C). This observation suggests that mitochondrial accumulation in the cytoplasm may be associated with impaired autophagic degradation. Based on this, we propose that mitochondrial relocalization under starvation conditions serves as a prelude to autophagic degradation.

### Structural similarity between TtATG8A and TtATG8B

**Fig 6:**
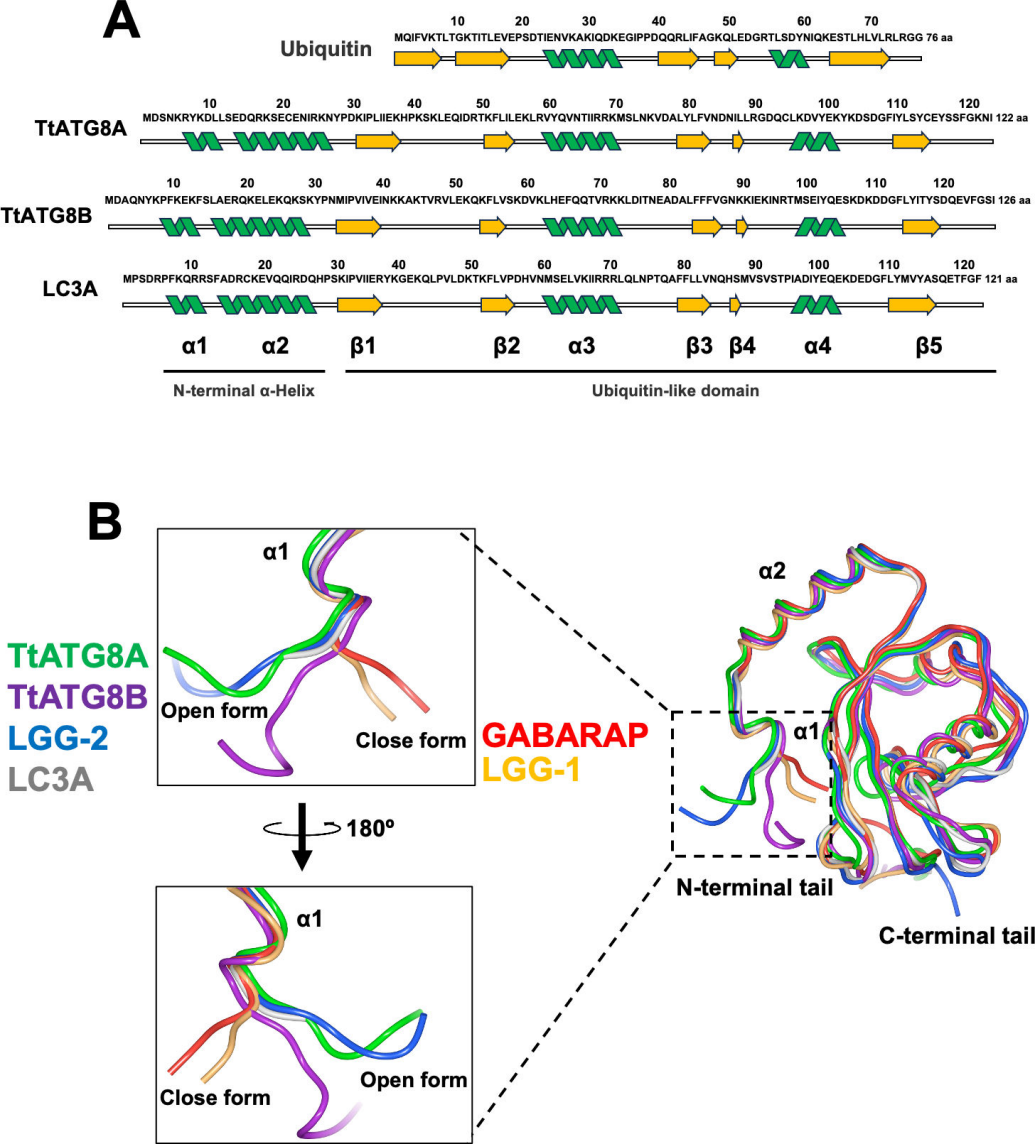
Structural comparison of ATG8 homologs. (**A**) Alignment of the secondary structures of ubiquitin (PDB:1UBI), TtATG8A (AlphaFoldDB: AF-I7LUK8-F1), TtATG8B (AlphaFoldDB: AF-Q22M73-F1), and LC3A (PDB:3WAL). (**B**) Tertiary structures of TtATG8A (AlphaFoldDB: AF-I7LUK8-F1), TtATG8B (AlphaFoldDB: AF-Q22M73-F1), LC3A (PDB:3WAL), GABARAP (PDB:1GNU), LGG-1 (PDB:5AZF), and LGG-2 (PDB:5AZH). The protein structures were visualized using Waals software (Altif Laboratories). Green: TtATG8A; purple: TtATG8B; blue: LGG-2; gray: LC3A; red: GABARAP; and yellow: LGG-1.

Structural predictions using AlphaFold2 reveal that TtATG8A and TtATG8B exhibit highly similar topologies (Fig. 6). Both proteins are composed of two N-terminal α-helices (α1 and α2), a ubiquitin-like domain consisting of multilayered β-sheets (β1–β5), and two additional α-helices (α3 and α4) (Fig. 6A). This structural arrangement aligns with the known structures of ATG8 homologs as determined by X-ray crystallography and NMR (28, 29). To explore potential functional differences between TtATG8A and TtATG8B, we examined their N-terminal tail conformations. The N-terminal α-helices of ATG8 play a crucial role in membrane tethering, hemifusion, and expansion (30–32). These conformations are classified into two distinct forms, open form (O-form) and closed form (C-form). The C-form, which includes members like GABARAP and LGG-1, is known to have higher membrane tethering and fusion activities compared to the O-form (represented by LC3A and LGG-2) (33, 34). Our simulations indicate that both TtATG8A and TtATG8B adopt an O-form conformation (Fig. 6B), similar to LGG-2 and LC3A. This contrasts with the C-form conformation seen in LGG-1 and GABARAP. The similarity in N-terminal conformation between TtATG8A and TtATG8B suggests that both proteins may share comparable abilities in autophagosome formation, contributing similarity to the mitochondrial degradation process.

## DISCUSSION

**Fig 7:**
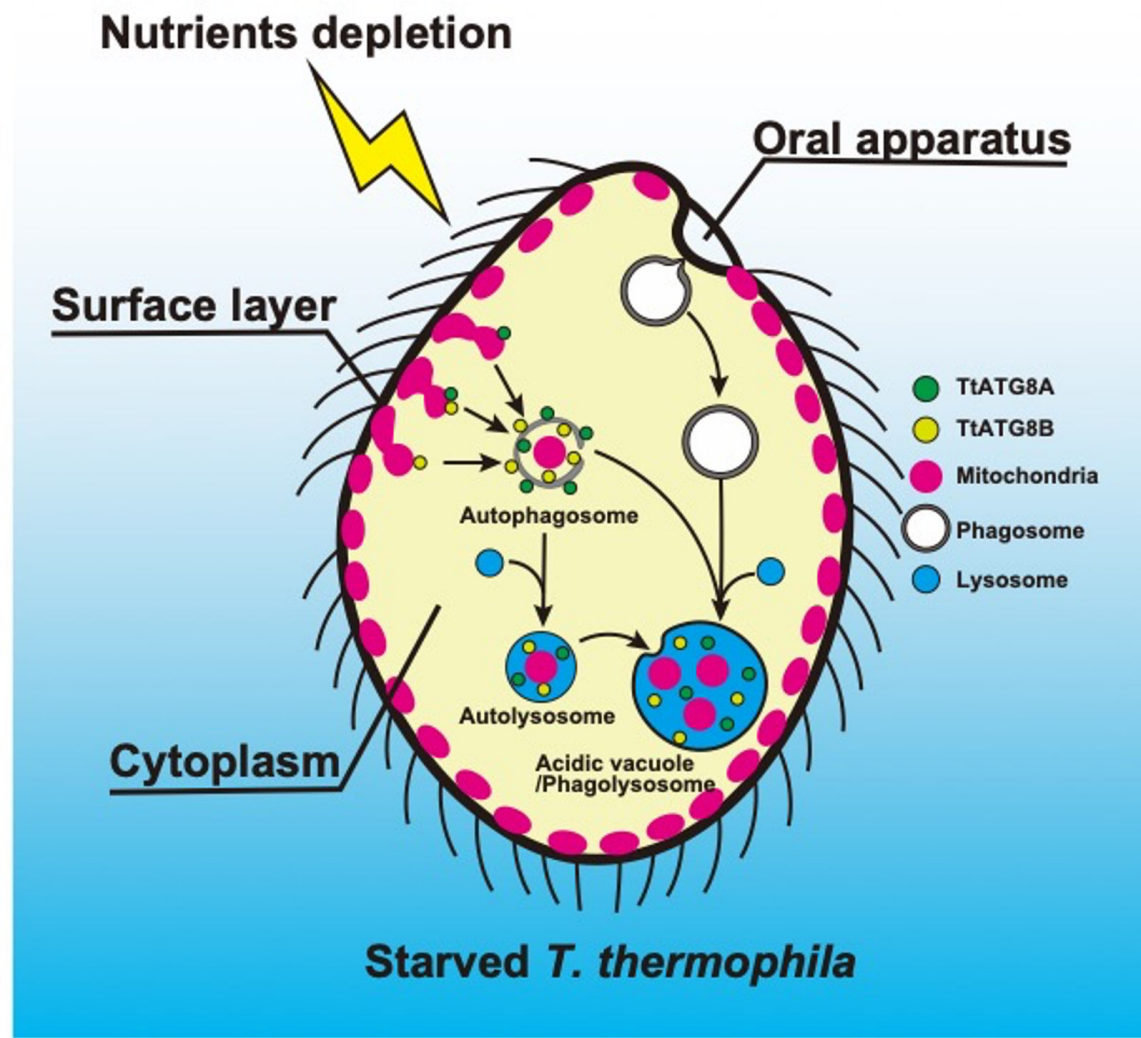
Schematic diagram of mitochondrial degradation by two ATG8 homologs in starved *T. thermophila*. In response to nutrient depletion, TtATG8A and TtATG8B interact with mitochondria, leading to the formation of autophagosomes. These autophagosomes then fuse with lysosomes to form autolysosomes, where the mitochondria associated with TtATG8A or TtATG8B are eventually degraded. Based on previous reports of mitochondrial remnants accumulating within vacuoles (4, 7, 8) and fluorescence microscopy observations (see Fig. 1; Fig. S1B), some autophagosomes containing mitochondria are also transported to acidic vacuoles (phagolysosomes). As a prelude to autophagic degradation, cortical mitochondria under starvation conditions may relocalize to the cytoplasm. Further details are discussed in the text.

In this study, we have demonstrated that two of the five ATG8 homologs, TtATG8A and TtATG8B, are involved in the degradation of mitochondria in *T. thermophila* cells under nutrient-depleted conditions. The transcription levels of *TtATG8A* and *TtATG8B* increased immediately after starvation (Fig. S3 and S4). These proteins generally appeared independently in granular form in the cytoplasm and eventually made contact with mitochondria (Fig. 3E-ii). Both proteins were also observed forming cup-like structures that enveloped mitochondria. Moreover, CLEM revealed that TtATG8A and TtATG8B localized to partially degraded mitochondria encased by a presumed autophagosome membrane. No specific hierarchical relationship was observed in the localization of these two proteins. Additionally, experiments involving gene expression shutoff for *TtATG8A* and *TtATG8B* both resulted in defects in mitochondrial degradation. Taken together, these results suggest that while TtATG8A and TtATG8B may act independently in mitochondrial degradation, both are necessary for efficient mitochondrial degradation (Fig. 7).

Although the structural features of TtATG8A and TtATG8B are highly similar (Fig. 6), *TtATG8B* is generally transcribed at higher levels than *TtATG8A*, with this difference becoming even more pronounced during starvation (Fig. S4). Interestingly, a previous study found that knockout of *TtATG8A* (*ATG8-65*) led to a rapid decrease in cell viability after a few days of nutrient depletion, while knockout of *TtATG8B* (*ATG8-2*) had no significant effect on cell viability under the same conditions (14). This suggests that TtATG8A is more crucial for cell viability in starved cells, despite its lower expression compared to TtATG8B. In mammalian cells, there is believed to be minimal functional redundancy among the LC3/GABARAP family proteins due to differences in their interacting proteins and transcription factors (35). Therefore, TtATG8A and TtATG8B may regulate mitochondrial degradation at different steps and conditions. In addition, there may be differences in the efficiency of PE conjugation and its turnover in these proteins (Fig. 3C and D). In the future, if antibodies that can distinguish and detect endogenous TtATG8A and TtATG8B can be produced, it may be possible to elucidate the differences in their intracellular behavior in detail.

Notably, we observed that the mitochondrial density in *TtATG8A* knockdown cells was higher than in wild-type and *TtATG8B* knockdown cells, even before starvation induction (see cells in vegetative growth condition in Fig. 5). This suggests that TtATG8A might play a role in mitochondrial degradation even during vegetative growth. Homeostatic mitochondrial degradation, also known as basal mitophagy, is crucial for maintaining cellular health by eliminating dysfunctional mitochondria, as demonstrated in yeasts and animal cells (16). Additionally, deficiencies in mitophagy can lead to excessive production of reactive oxygen species, mitochondrial DNA damage, and reduced respiratory capacity during nitrogen starvation in *S. cerevisiae* (36). Therefore, TtATG8A may be more important than TtATG8B for maintaining homeostasis by eliminating damaged mitochondria in vegetative cells. It is possible that cells deficient in TtATG8A exhibit severe negative effects on cell survival under starvation conditions due to an accumulation of unhealthy mitochondria. Future research should focus on the quality control and quantity of mitochondria to understand these dynamics.

### Selective mitochondrial degradation in *Tetrahymena*

According to pioneering research by Aufderheide (4), mitochondria on the cell surface can be broadly categorized into two types based on their spatial arrangement relative to the basal body: those attached to the transverse microtubules and those located on the opposite side of the basal body, aligning with the longitudinal microtubules. The former type of mitochondrial arrangement is observed in all cells, while the latter is not consistently present across all cells. Notably, the latter type of mitochondria is no longer observed in all cells within 3 h after starvation. This suggests that mitochondria near the longitudinal microtubules might be separated from the cell surface by some mechanism, after which ATG8 could act on them to induce degradation. The localized modification of mitochondria specifically associated with longitudinal microtubules may contribute to the efficient reduction of cortical mitochondria in starved *Tetrahymena*. Indeed, microtubule-dependent motor proteins are known to play a crucial role in mitochondrial transport across various species (37). Thus, it is possible that kinesin or dynein in *Tetrahymena* possibly contributes to the transport of cortical mitochondria into the cytoplasm. Further investigation into the relationship between mitochondria and microtubules will be essential for understanding how *Tetrahymena* efficiently removes cortical mitochondria during starvation. Alternatively, another hypothesis is that starvation-induced shrinkage of *Tetrahymena* cells may physically push cortical mitochondria away from the cell surface.

CLEM analysis revealed that autophagosomes labeled with TtATG8A or TtATG8B contained no notable structures other than mitochondria (Fig. 4; Fig. S11). On the other hand, ER-like structures were often found around autophagosomes (Fig. 4). This suggests that TtATG8A and TtATG8B may specifically recognize mitochondria released from the cell surface. Two primary pathways, adaptor- and ubiquitin-dependent autophagy, are involved in the selective degradation of mitochondria during mitophagy (16). In the adaptor-dependent pathway, during nitrogen starvation, autophagy adaptor proteins, such as ScAtg32 (in *S. cerevisiae*), SpAtg43 (in *Schizosaccharomyces pombe*), NIX, and BNIP3 (in mammals) interact with Atg8 to induce mitochondrial sequestration by the isolation membrane. These adaptor proteins, which are single-pass transmembrane proteins in the outer mitochondrial membrane, facilitate mitochondrial degradation (38–42). In contrast, ubiquitin-dependent mitophagy, which contributes to mitochondrial homeostasis in metazoans, such as worms, flies, and mammals, involves the PINK1/parkin pathway. This pathway is critical for sensing and ubiquitinating damaged mitochondria. Ubiquitinated mitochondria are then targeted by adaptor proteins and ATG8 and are sequestered by an isolation membrane (43). While these pathways are well documented in various species, little is known about mitophagy mechanisms in eukaryotes other than Opisthokonta. Unfortunately, BLAST searches did not identify *Tetrahymena* orthologs for the yeast mitophagy receptors ScAtg32 and SpAtg43, or the mammalian mitophagy receptors NIX and BNIP3. This suggests that *Tetrahymena* might possess a unique mitophagy receptor. To elucidate how TtATG8A and TtATGB selectively recognize mitochondria for degradation, it will be crucial to investigate the differences in composition and physiological state between cortical and cytoplasmic mitochondria.

### Phagosomes may contribute to the bulk degradation of mitochondria

*Tetrahymena* possesses an elaborate membrane trafficking system that enables the rapid phagocytosis of bacterial-sized particles. In this study, we found that mitochondria accumulated in FITC-dextran-labeled phagosomes (Fig. S1B). This finding suggests that *Tetrahymena* likely utilizes phagosomes or phagolysosomes to collect mitochondria destined for degradation, allowing their bulk degradation. It has recently been reported that autophagosomes fuse with phagosomes to efficiently digest apoptotic dead cells in *Caenorhabditis elegans* (44). In starved *Tetrahymena*, active fusion of autophagosomes and phagosomes or phagolysosomes may occur to rapidly and efficiently degrade mitochondria.

The fusion of lysosomes and autophagosomes, as well as lysosomes and phagosomes, is known to be mediated by Rab7, a member of the Rab GTPase family, and homotypic fusion and vacuole protein sorting complex (HOPS) as a membrane tethering protein (45, 46). This mechanism is also adopted in the fusion of autophagosomes and phagosomes in *C. elegans* mentioned above. However, HOPS-specific subunits (Vps39 and Vps41) are absent in ciliates (47), and class C core vacuole/endosome transport (CORVET) and Rab7 replace the lost HOPS function in *T. thermophila* (47, 48). Therefore, the autophagosomes-phagosomes fusion is expected to be promoted by the CORVET/Rab7 complex in *Tetrahymena*. Alternatively, there are a number of *rab* genes of unknown function in *T. thermophila* (49, 50), and it is possible that any of these gene products and their target proteins are involved in autophagosome-phagosome fusion during starvation. Hence, functional analysis of the CORVET/Rab7 complex and other Rab proteins in starved *Tetrahymena* would advance our comprehension of the crosstalk between autophagy and phagocytosis.

## MATERIALS AND METHODS

### *Tetrahymena* strains and culture conditions

The strains used in this study are detailed in Table S1. Cells were cultured in super proteose peptone (SPP) medium, containing 1% proteose peptone, 0.2% glucose, 0.1% yeast extract, and 0.003% EDTA-ferric sodium salt, along with antibiotics (70 µg/mL penicillin and 100 µg/mL streptomycin) (51), at 30°C until reaching the log phase (10^4^–10^6^ cells/mL). Nutrient starvation was achieved by washing the cells and resuspending them in 10 mM Tris-HCl (pH 7.5). The starved cells were then incubated statically at 30°C.

### Construction of plasmids

Plasmid vectors and primers used in this study are shown in Fig. S13 and S14 and Table S2. To generate cell strains expressing EGFP- or mCherry-fused TtATG8s at their N-terminus, cassettes containing *Neo4* (52), the *MTT2* promoter (*P_MTT2_*) (22), and a fluorescent protein gene were inserted at the 5′ end of the *TtATG8s* open reading frame using the In-fusion HD Cloning Kit (Takara). Details are provided in the supplemental figure legends (Fig. S13). The resulting plasmids, named pT7Blue-*P_MTT1_-Neo4-P_MTT2_-EGFP-TtATG8s* and pT7Blue-*P_MTT1_-Pur4-P_MTT2_-mCherry-TtATG8B* (Fig. S14), were verified through restriction enzyme and sequence analyses.

### Transformation of *T. thermophila*

Transformation of *T. thermophila* was carried out as previously described (53). Once *T. thermophila* B2086 cells reached the log phase (10^4^–10^6^ cells/mL), the culture medium was replaced with 10 mM Tris-HCl (pH 7.5) and incubated overnight at 30°C. Targeting plasmids were digested with SalI and KpnI to remove the plasmid backbone, coated with gold particles (0.6 µm diameter, Bio-Rad), and introduced into the cells using a biolistic gun PSD-1000/He (Bio-Rad) equipped with a 900 psi rupture disk. The transformed cells were then selected in SPP medium containing 1 µg/mL CdCl_2_, 100 µg/mL paromomycin, or 200 µg/mL puromycin. Given that the macronucleus of *Tetrahymena* is polyploid, the number of *EGFP-TtATG8s* (or *mCherry-TtATG8B*) copies is increased through phenotypic assortment (54). Finally, viable transformed cells were isolated from SPP medium containing 10^−9^ µg/mL CdCl_2_ and 600 µg/mL paromomycin or 1.2 mg/mL puromycin. Gene recombination and protein expression were confirmed by PCR and immunoblotting, respectively (Fig. S15 to S20).

### Protein preparation, immunoprecipitation, and immunoblotting

Cultured cells were centrifuged, and the resulting pellets were resuspended in lysis buffer composed of 50 mM Tris-HCl (pH 7.5), 150 mM NaCl, 10% glycerol, 0.2% NP-40, 10 mM β-glycerophosphate, 10 mM para-nitrophenyl phosphate, 10 mM NaF, 20 µg/mL leupeptin, 40 µg/mL aprotinin, and 1 mM phenylmethylsulfonyl fluoride. The lysates, subjected to freeze-thaw cycles, were centrifuged, and the supernatant was mixed with SDS sample buffer containing 62.5 mM Tris-HCl (pH 6.8), 1% SDS, 10% glycerol, 0.002% bromophenol blue, and 10% 2-mercaptoethanol, then boiled for 5 min. The boiled extracts were loaded onto SDS-PAGE containing 6 M urea. Immunoblotting was carried out using anti-mouse GFP (Roche), anti-α-tubulin (Wako), and anti-TAT1 antibodies (55). Following incubation with horseradish peroxidase (HRP)-conjugated secondary antibodies, proteins were detected using Western BLoT Quant HRP Substrate (Takara) and an AE-9300H Ez-Capture MG image analyzer (ATTO).

For proteins with low extraction efficiency, immunoprecipitation was employed to enhance detection. Cell pellets were lysed in the same lysis buffer, and the freeze-thawed lysates were centrifuged. The supernatants were incubated with protein G-Sepharose (GE Healthcare) and anti-mouse GFP antibody (Roche) overnight at 4°C. The immunoprecipitants were washed three times with lysis buffer, then boiled with SDS sample buffer, separated by SDS-PAGE, and subjected to immunoblotting using anti-mouse GFP antibody (Roche).

### Fluorescent staining and microscopy

To visualize mitochondria, cells were treated with 200 µM MitoTracker Red CMXRos (Thermo Fisher Scientific), 200 nM MitoTracker Deep Red FM (Thermo Fisher Scientific), or 0.1 µM MitoBright LT Green (Dojindo) for 30–60 min. For staining lysosomes or vacuoles, cells were incubated with 1:2,000 diluted LysoPrime Green (Dojindo), 1:1,000 diluted pHLys Red (Dojindo), and 0.1 µM LysoTracker Red DND-99 (Thermo Fisher Scientific) for 30–60 min. The ER was labeled by incubating cells with 1 µM ER-Tracker Blue-White DPX (Thermo Fisher Scientific) for 1 h. FITC-Dextran was obtained from Adooq Bioscience. Chemical fixation was performed using 2% paraformaldehyde in 20 mM phosphate buffer (pH 7.5) for 30 min. For live cell observation, cells were placed on a slide glass, and a 0.053 mm diameter fishing line (TRAY) was inserted between the slide glass and the cover glass to pinch and secure the cells. Fluorescence images were captured using a BX51 (Evident) or TSC SP8 confocal microscope (Leica Microsystems). Deconvolution of fluorescence microscopy images was performed using a Thunder imaging system (Leica Microsystems). We analyzed the fluorescence signal of TtATG8 proteins fused with EGFP or mCherry as granules in which the intensity of the signal was clearly distinguishable from the background, and the diameter was larger than 0.5 µm.

### Correlative light and electron microscopy

CLEM was performed as previously described (56). Briefly, *T. thermophila* cells expressing *EGFP-TtATG8A* or *EGFP-TtATG8B* were cultured overnight in SPP containing 1.5 mM CuSO_4_. The cells were then starved for 2.5 h and stained with 200 nM MitoTracker Red CM-H2Xros for 30 min. After staining, the cells were washed twice with 20 mM phosphate buffer (pH 7.5) and fixed in a primary fixative solution consisting of 20 mM phosphate buffer (pH 7.5), 2% paraformaldehyde, and 0.5% glutaraldehyde for 30 min. The fixed cells were washed with 20 mM phosphate buffer and placed onto a glass bottom dish with 150 µm grids (TCI-3922-035R-1CS; Iwaki, a custom-made product with cover glass attached in the opposite direction) coated with carbon and poly-L-lysine. Approximately 20 Z-stack images of the cells were acquired at 0.35 µm intervals from the cell surface to the interior using an FV3000 confocal microscope (Evident). The cells were then fixed overnight with 2.5% glutaraldehyde in 0.1 M sodium cacodylate buffer at 4°C. Following this, the cells were washed with 0.1 M sodium cacodylate buffer, treated with ferrocyanide-reduced osmium tetroxide (1% OsO_4_, 1.5% K_4_[Fe(CN)_6_], and 0.065 M sodium cacodylate buffer) at 4°C for 2 h, and rinsed five times with Milli-Q water. The samples were en bloc stained with 2% uranyl acetate solution and rinsed five times with Milli-Q water. Dehydration was carried out using an ethanol series, and the samples were embedded in EPON812 (TAAB). After polymerization, the cover glasses were removed by soaking in liquid nitrogen. The sample blocks were trimmed to approximately the same size as the fluorescent images (approximately 150 × 150 µm). Serial sections (25 nm thick) were cut using an ultramicrotome (UC7; Leica Microsystems) and a diamond knife with a large boat (Ultrajumbo, 35° or 45°, Diatome). The sections were collected on a pre-cleaned silicon wafer strip and examined under a SEM (JSM-7900F; JEOL). CLEM images were constructed using Fiji software (NIH).

### Statistical analysis

Data are presented as the mean ± s.e.m of the mean from the indicated number of observations. To determine significant differences, Welch’s *t*-test (unequal variance assumed) and one-way analysis of variance followed by Tukey’s multiple comparison test were conducted using Prism version 8. Statistical significance was set at *P <* 0.05.

## SUPPLEMENTAL MATERIAL

Supplemental material mbio.00783-25-s0001.pdf Fig. S1 to S20; Tables S1 and S2.

ASM does not own the copyrights to Supplemental Material that may be linked to, or accessed through, an article. The authors have granted ASM a non-exclusive, world-wide license to publish the Supplemental Material files. Please contact the corresponding author directly for reuse.

## References

[1] Kiel JAKW. 2010. Autophagy in unicellular eukaryotes. Phil Trans R Soc B 365:819–830. doi:10.1098/rstb.2009.0237 20124347 PMC2817228

[2] Kourtis N, Tavernarakis N. 2009. Autophagy and cell death in model organisms. Cell Death Differ 16:21–30. doi:10.1038/cdd.2008.120 19079286

[3] Cole E, Gaertig J. 2022. Anterior-posterior pattern formation in ciliates. J Eukaryot Microbiol 69:e12890. doi:10.1111/jeu.12890 35075744 PMC9309198

[4] Aufderheide K. 1979. Mitochondrial associations with specific microtubular components of the cortex of Tetrahymena thermophila. I. Cortical patterning of mitochondria. J Cell Sci 39:299–312. doi:10.1242/jcs.39.1.299 528586

[5] Nelsen EM. 1978. Transformation in Tetrahymena thermophila. development of an inducible phenotype. Dev Biol 66:17–31. doi:10.1016/0012-1606(78)90270-1 109331

[6] Nelsen EM, Debault LE. 1978. Transformation in Tetrahymena pyriformis: description of an inducible phenotype. J Protozool 25. doi:10.1111/j.1550-7408.1978.tb03880.x 96253

[7] Levy MR, Elliott AM. 1968. Biochemical and ultrastructural changes in Tetrahymena pyriformis during starvation. J Protozool 15. doi:10.1111/j.1550-7408.1968.tb02113.x 5643475

[8] Nilsson JR. 1984. On starvation-induced autophagy in Teraahymena. Carlsberg Res Commun 49:323–340. doi:10.1007/BF02913960

[9] Yamamoto H, Zhang S, Mizushima N. 2023. Autophagy genes in biology and disease. Nat Rev Genet 24. doi:10.1038/s41576-022-00562-w PMC9838376 36635405

[10] Klionsky DJ, Cuervo AM, Seglen PO. 2007. Methods for monitoring autophagy from yeast to human. Autophagy 3:181–206. doi:10.4161/auto.3678 17224625

[11] Mizushima N. 2020. The ATG conjugation systems in autophagy. Curr Opin Cell Biol 63:1–10. doi:10.1016/j.ceb.2019.12.001 31901645

[12] Matoba K, Noda NN. 2021. Structural catalog of core Atg proteins opens new era of autophagy research. J Biochem 169:517–525. doi:10.1093/jb/mvab017 33576807

[13] Gatica D, Lahiri V, Klionsky DJ. 2018. Cargo recognition and degradation by selective autophagy. Nat Cell Biol 20:233–242. doi:10.1038/s41556-018-0037-z 29476151 PMC6028034

[14] Liu M-L, Yao M-C. 2012. Role of ATG8 and autophagy in programmed nuclear degradation in Tetrahymena thermophila. Eukaryot Cell 11:494–506. doi:10.1128/EC.05296-11 22366125 PMC3318292

[15] Zhang S, Yazaki E, Sakamoto H, Yamamoto H, Mizushima N. 2022. Evolutionary diversification of the autophagy-related ubiquitin-like conjugation systems. Autophagy 18:2969–2984. doi:10.1080/15548627.2022.2059168 35427200 PMC9673942

[16] Onishi M, Yamano K, Sato M, Matsuda N, Okamoto K. 2021. Molecular mechanisms and physiological functions of mitophagy. EMBO J 40:e104705. doi:10.15252/embj.2020104705 33438778 PMC7849173

[17] Neikirk K, Marshall AG, Kula B, Smith N, LeBlanc S, Hinton A. 2023. MitoTracker: a useful tool in need of better alternatives. Eur J Cell Biol 102:151371. doi:10.1016/j.ejcb.2023.151371 37956476

[18] Wloga D, Strzyzewska-Jówko I, Gaertig J, Jerka-Dziadosz M. 2008. Septins stabilize mitochondria in Tetrahymena thermophila. Eukaryot Cell 7:1373–1386. doi:10.1128/EC.00085-08 18586950 PMC2519767

[19] Elliott AM, Clemmons GL. 1966. An ultrastructural study of ingestion and digestion in Tetrahymena pyriformis. J Protozool 13:311–323. doi:10.1111/j.1550-7408.1966.tb01912.x 5953849

[20] Tiedtke A, Kiy T, Vosskühler C, Rasmussen L. 1993. Pathways of lysosomal enzyme secretion in *Tetrahymena*, p 99–122. In Membrane traffic in protozoa. JAI Press, Greenwich, CT.

[21] Bo T, Kang Y, Liu Y, Xu J, Wang W. 2021. Atg5 regulates selective autophagy of the parental macronucleus during Tetrahymena sexual reproduction. Cells 10:3071. doi:10.3390/cells10113071 34831293 PMC8623110

[22] Boldrin F, Santovito G, Formigari A, Bisharyan Y, Cassidy-Hanley D, Clark TG, Piccinni E. 2008. MTT2, a copper-inducible metallothionein gene from Tetrahymena thermophila. Comp Biochem Physiol C Toxicol Pharmacol 147:232–240. doi:10.1016/j.cbpc.2007.10.002 18068524

[23] Cheong H, Klionsky DJ. 2008. Biochemical methods to monitor autophagy‐related processes in yeast. Methods Enzymol 451:1–26. doi:10.1016/S0076-6879(08)03201-1 19185709

[24] Besteiro S, Brooks CF, Striepen B, Dubremetz J-F. 2011. Autophagy protein Atg3 is essential for maintaining mitochondrial integrity and for normal intracellular development of Toxoplasma gondii tachyzoites. PLoS Pathog 7:e1002416. doi:10.1371/journal.ppat.1002416 22144900 PMC3228817

[25] Kirisako T, Ichimura Y, Okada H, Kabeya Y, Mizushima N, Yoshimori T, Ohsumi M, Takao T, Noda T, Ohsumi Y. 2000. The reversible modification regulates the membrane-binding state of Apg8/Aut7 essential for autophagy and the cytoplasm to vacuole targeting pathway. J Cell Biol 151:263–276. doi:10.1083/jcb.151.2.263 11038174 PMC2192639

[26] Tomlins AM, Ben-Rached F, Williams RA, Proto WR, Coppens I, Ruch U, Gilberger TW, Coombs GH, Mottram JC, Müller S, Langsley G. 2013. Plasmodium falciparum ATG8 implicated in both autophagy and apicoplast formation. Autophagy 9:1540–1552. doi:10.4161/auto.25832 24025672

[27] Kitamura K, Kishi-Itakura C, Tsuboi T, Sato S, Kita K, Ohta N, Mizushima N. 2012. Autophagy-related Atg8 localizes to the apicoplast of the human malaria parasite Plasmodium falciparum. PLoS One 7:e42977. doi:10.1371/journal.pone.0042977 22900071 PMC3416769

[28] Kumeta H, Watanabe M, Nakatogawa H, Yamaguchi M, Ogura K, Adachi W, Fujioka Y, Noda NN, Ohsumi Y, Inagaki F. 2010. The NMR structure of the autophagy-related protein Atg8. J Biomol NMR 47:237–241. doi:10.1007/s10858-010-9420-1 20428927

[29] Sugawara K, Suzuki NN, Fujioka Y, Mizushima N, Ohsumi Y, Inagaki F. 2004. The crystal structure of microtubule-associated protein light chain 3, a mammalian homologue of Saccharomyces cerevisiae Atg8. Genes Cells 9:611–618. doi:10.1111/j.1356-9597.2004.00750.x 15265004

[30] Nakatogawa H, Ichimura Y, Ohsumi Y. 2007. Atg8, a ubiquitin-like protein required for autophagosome formation, mediates membrane tethering and hemifusion. Cell 130:165–178. doi:10.1016/j.cell.2007.05.021 17632063

[31] Weidberg H, Shpilka T, Shvets E, Abada A, Shimron F, Elazar Z. 2011. LC3 and GATE-16 N termini mediate membrane fusion processes required for autophagosome biogenesis. Dev Cell 20:444–454. doi:10.1016/j.devcel.2011.02.006 21497758

[32] Zhang W, Nishimura T, Gahlot D, Saito C, Davis C, Jefferies HBJ, Schreiber A, Thukral L, Tooze SA. 2023. Autophagosome membrane expansion is mediated by the N-terminus and cis-membrane association of human ATG8s. Elife 12:e89185. doi:10.7554/eLife.89185 37288820 PMC10289813

[33] Landajuela A, Hervás JH, Antón Z, Montes LR, Gil D, Valle M, Rodriguez JF, Goñi FM, Alonso A. 2016. Lipid geometry and bilayer curvature modulate LC3/GABARAP-mediated model autophagosomal elongation. Biophys J 110:411–422. doi:10.1016/j.bpj.2015.11.3524 26789764 PMC4724631

[34] Wu F, Watanabe Y, Guo X-Y, Qi X, Wang P, Zhao H-Y, Wang Z, Fujioka Y, Zhang H, Ren J-Q, Fang T-C, Shen Y-X, Feng W, Hu J-J, Noda NN, Zhang H. 2015. Structural basis of the differential function of the two C. elegans Atg8 homologs, LGG-1 and LGG-2, in autophagy. Mol Cell 60:914–929. doi:10.1016/j.molcel.2015.11.019 26687600

[35] Schaaf MBE, Keulers TG, Vooijs MA, Rouschop KMA. 2016. LC3/GABARAP family proteins: autophagy-(un)related functions. FASEB J 30:3961–3978. doi:10.1096/fj.201600698R 27601442

[36] Kurihara Y, Kanki T, Aoki Y, Hirota Y, Saigusa T, Uchiumi T, Kang D. 2012. Mitophagy plays an essential role in reducing mitochondrial production of reactive oxygen species and mutation of mitochondrial DNA by maintaining mitochondrial quantity and quality in yeast. J Biol Chem 287:3265–3272. doi:10.1074/jbc.M111.280156 22157017 PMC3270981

[37] Kruppa AJ, Buss F. 2021. Motor proteins at the mitochondria-cytoskeleton interface. J Cell Sci 134:jcs226084. doi:10.1242/jcs.226084 33912943 PMC8077471

[38] Fukuda T, Ebi Y, Saigusa T, Furukawa K, Yamashita S-I, Inoue K, Kobayashi D, Yoshida Y, Kanki T. 2020. Atg43 tethers isolation membranes to mitochondria to promote starvation-induced mitophagy in fission yeast. Elife 9:e61245. doi:10.7554/eLife.61245 33138913 PMC7609059

[39] Hanna RA, Quinsay MN, Orogo AM, Giang K, Rikka S, Gustafsson ÅB. 2012. Microtubule-associated protein 1 light chain 3 (LC3) interacts with Bnip3 protein to selectively remove endoplasmic reticulum and mitochondria via autophagy. J Biol Chem 287:19094–19104. doi:10.1074/jbc.M111.322933 22505714 PMC3365942

[40] Kanki T, Wang K, Cao Y, Baba M, Klionsky DJ. 2009. Atg32 is a mitochondrial protein that confers selectivity during mitophagy. Dev Cell 17:98–109. doi:10.1016/j.devcel.2009.06.014 19619495 PMC2746076

[41] Novak I, Kirkin V, McEwan DG, Zhang J, Wild P, Rozenknop A, Rogov V, Löhr F, Popovic D, Occhipinti A, Reichert AS, Terzic J, Dötsch V, Ney PA, Dikic I. 2010. Nix is a selective autophagy receptor for mitochondrial clearance. EMBO Rep 11:45–51. doi:10.1038/embor.2009.256 20010802 PMC2816619

[42] Okamoto K, Kondo-Okamoto N, Ohsumi Y. 2009. Mitochondria-anchored receptor Atg32 mediates degradation of mitochondria via selective autophagy. Dev Cell 17:87–97. doi:10.1016/j.devcel.2009.06.013 19619494

[43] Uoselis L, Nguyen TN, Lazarou M. 2023. Mitochondrial degradation: mitophagy and beyond. Mol Cell 83:3404–3420. doi:10.1016/j.molcel.2023.08.021 37708893

[44] Peña-Ramos O, Chiao L, Liu X, Yu X, Yao T, He H, Zhou Z. 2022. Autophagosomes fuse to phagosomes and facilitate the degradation of apoptotic cells in Caenorhabditis elegans Elife 11:e72466. doi:10.7554/eLife.72466 34982028 PMC8769646

[45] Nguyen JA, Yates RM. 2021. Better together: current insights into phagosome-lysosome fusion. Front Immunol 12:636078. doi:10.3389/fimmu.2021.636078 33717183 PMC7946854

[46] Yim WW-Y, Mizushima N. 2020. Lysosome biology in autophagy. Cell Discov 6:6. doi:10.1038/s41421-020-0141-7 32047650 PMC7010707

[47] Sparvoli D, Richardson E, Osakada H, Lan X, Iwamoto M, Bowman GR, Kontur C, Bourland WA, Lynn DH, Pritchard JK, Haraguchi T, Dacks JB, Turkewitz AP. 2018. Remodeling the specificity of an endosomal CORVET tether underlies formation of regulated secretory vesicles in the ciliate Tetrahymena thermophila. Curr Biol 28:697–710. doi:10.1016/j.cub.2018.01.047 29478853 PMC5840023

[48] Sparvoli Daniela, Zoltner M, Cheng C-Y, Field MC, Turkewitz AP. 2020. Diversification of CORVET tethers facilitates transport complexity in Tetrahymena thermophila J Cell Sci 133:jcs238659. doi:10.1242/jcs.238659 31964712 PMC7033735

[49] Bright LJ, Kambesis N, Nelson SB, Jeong B, Turkewitz AP. 2010. Comprehensive analysis reveals dynamic and evolutionary plasticity of Rab GTPases and membrane traffic in Tetrahymena thermophila. PLoS Genet 6:e1001155. doi:10.1371/journal.pgen.1001155 20976245 PMC2954822

[50] Saito-Nakano Y, Nakahara T, Nakano K, Nozaki T, Numata O. 2010. Marked amplification and diversification of products of ras genes from rat brain, Rab GTPases, in the ciliates Tetrahymena thermophila and Paramecium tetraurelia. J Eukaryot Microbiol 57:389–399. doi:10.1111/j.1550-7408.2010.00503.x 20738463

[51] Gaertig J, Wloga D, Vasudevan KK, Guha M, Dentler W. 2013. Discovery and functional evaluation of ciliary proteins in Tetrahymena thermophila. Methods Enzymol 525:265–284. doi:10.1016/B978-0-12-397944-5.00013-4 23522474 PMC4392907

[52] Mochizuki K. 2008. High efficiency transformation of Tetrahymena using a codon-optimized neomycin resistance gene. Gene 425:79–83. doi:10.1016/j.gene.2008.08.007 18775482

[53] Kushida Y, Takaine M, Nakano K, Sugai T, Vasudevan KK, Guha M, Jiang Y-Y, Gaertig J, Numata O. 2017. Kinesin-14 is important for chromosome segregation during mitosis and meiosis in the ciliate Tetrahymena thermophila. J Eukaryot Microbiol 64:293–307. doi:10.1111/jeu.12366 27595611

[54] Ruehle MD, Orias E, Pearson CG. 2016. Tetrahymena as a unicellular model eukaryote: genetic and genomic tools. Genetics 203:649–665. doi:10.1534/genetics.114.169748 27270699 PMC4896184

[55] Woods A, Sherwin T, Sasse R, MacRae TH, Baines AJ, Gull K. 1989. Definition of individual components within the cytoskeleton of Trypanosoma brucei by a library of monoclonal antibodies. J Cell Sci 93 (Pt 3):491–500. doi:10.1242/jcs.93.3.491 2606940

[56] Takahashi S, Saito C, Koyama-Honda I, Mizushima N. 2022. Quantitative 3D correlative light and electron microscopy of organelle association during autophagy. Cell Struct Funct 47:89–99. doi:10.1247/csf.22071 36418108 PMC10511054

