# Supplementary material for: Tetrahymena ATG8 homologs, TtATG8A and TtATG8B, are responsible for mitochondrial degradation induced by starvation
Source: mBio. 2025 May 15;16(6):e00783-25. doi: 10.1128/mbio.00783-25 (PMC12153319; doi:10.1128/mbio.00783-25)
Supplement: Supplemental material — Fig. S1 to S20; Tables S1 and S2. [file mbio.00783-25-s0001.pdf]

Supplementary Materials and Methods

RNA Preparation and Quantitative PCR Analysis

Cultured cells were treated with or without 1.5 mM $CuSO_4$ overnight at 30°C. To induce nutrients starvation, cells were incubated in 10 mM Tris-HCl buffer (pH 7.5) for 3 hours at 30 °C. Total RNA was extracted from cell pellets using the FastGene RNA Premium Kit (NIPPON Genetics) according to the manufacturer's instructions. RNA concentration and purity were measured using a NanoDrop 1000 spectrophotometer (Thermo Fisher Scientific). Subsequently, 0.5 μg of total RNA was reverse transcribed into complementary DNA (cDNA) using the ReverTra Ace qPCR RT Master Mix with gDNA Remover (TOYOBO). Quantitative PCR (qPCR) was performed using SYBR Green qPCR Master Mix (Thermo Fisher Scientific) and the QuantStudio 5 Real-Time PCR System (Thermo Fisher Scientific). *BTU1* (*Beta Tubulin 1*) was employed as the internal standard. Relative gene expression levels were calculated using the ΔΔCt method. Schematic of RNA splicing and primer design for quantitative PCR analysis were described in Fig. S2. Primer sequences are described below. *TtATG8A*, forward primer 5′-TCCTGATAAGATTCCCCTCATTATT-3′, reverse primer 5′-AGAGAGCATCTACTTTATTGAGACT-3′; *TtATG8B*: forward primer 5′-CAAAAACAGTTAGAGTATTG-3′, reverse primer 5′-GATTCTTGGTAAATTTCGCT-3′. *BTU1*: forward primer 5′-CACTGTTCCTGAATTGACCT-3′, reverse primer 5′-AGCCATCTTAAGACCCTTAG-3′.

Supplementary Figure legends and Tables

TABLE S1. *T. thermophila* strains used in this study

No.	Strain	Selection maker
1	*B2086 Wild-type*	
2	*B2086 harboring P_{MTT2}-EGFP-TtATG8A*	*P_{MTT1}-Neo4*
3	*B2086 harboring P_{MTT2}-EGFP-TtATG8B*	*P_{MTT1}-Neo4*
4	*B2086 harboring P_{MTT2}-EGFP-TtATG8C*	*P_{MTT1}-Neo4*
5	*B2086 harboring P_{MTT2}-EGFP-TtATG8D*	*P_{MTT1}-Neo4*
6	*B2086 harboring P_{MTT2}-EGFP-TtATG8F*	*P_{MTT1}-Neo4*
7	*B2086 harboring P_{MTT2}-EGFP-TtATG8A, P_{MTT2}-mCherry-TtATG8B*	*P_{MTT1}-Neo4, P_{MTT1}-Pur4*

TABLE S2. Primers used in this study

No.	Primer Name	Sequence (5'→3')
1	ATG8A F1	5' - CCCGGGCTAGCTAGAAACGTTCTCATGTTCTGATC - 3'
2	ATG8A R1	5' - TGGCCATTAAAACTGCTAAAATATTTAGAATAGCC - 3'
3	ATG8A F2	5' - TCGAGGGGGGGCCCGGTAGAAATGGACTCAAATAAACG - 3'
4	ATG8A R2	5' - TATCGAATTCCTGCAGCCATGTCAACTTTCTTACCTTTTC - 3'
5	ATG8B F1	5' - CCCGGGATATAAGAAACTCACTTTAGTATAAGAGG - 3'
6	ATG8B R1	5' - TGGCCAATCTAGAGCAGATAATATATTGGTTGTTC - 3'
7	ATG8B F2	5' - TCGAGGGGGGGCCCGGTAATGGACGCTCAAAATTATAAACCC - 3'
8	ATG8B R2	5' - TATCGAATTCCTGCAGCCGTTGTTTACTTTTTGCCTTTATG - 3'
9	ATG8C F1	5' - GCCGGCTAAACCTACACTCAAAATGGTTATGGAAG - 3'
10	ATG8C R1	5' - CCCGGGTTTAGTTCATAAAACATTAATTCAACTGC - 3'
11	ATG8C F2	5' - TCGAGGGGGGGCCCGGTACATTTTAAAGTAATTCCAACAAG - 3'
12	ATG8C R2	5' - TATCGAATTCCTGCAGCCTATAAGCCAGTCAAATTCCTATC - 3'
13	ATG8D F1	5' - TGTACAGAGAATGACATAAGTAGATCCTAAGAAGC- 3'
14	ATG8D R1	5' - TGTACAGTTAATTAATAATTATCTAGAATCAAATG - 3'
15	ATG8D F2	5' - TCGAGGGGGGGCCCGGTAATGTTTAAAATTTGCAAAAAGAG - 3'
16	ATG8D R2	5' - TATCGAATTCCTGCAGCCATGCAATTTTGTGGTGCTTG - 3'
17	ATG8F F1	5' - TGGCCAGCTTTGCATTTTATTGGGTCTACTGATAT - 3'
18	ATG8F R1	5' - GCCGGCCAAGGCATACGATAATGACTTGTGGTTCC - 3'
19	ATG8F F2	5' - TCGAGGGGGGGCCCGGTAATGAATTAAACAGCAGAAAGC - 3'
20	ATG8F R2	5' - TATCGAATTCCTGCAGCCCGATATTACTTAAATATAAACGAC- 3'
21	Seq#1	5' - GGCTGCAGGAATTCGATA- 3'
22	Seq#2	5' - TACCGGGCCCCCCCTCGA- 3'
23	ATG8A check R	5' - TTTAAAAAAGAAAATCATCAATAATTAAGATTTAT - 3'
24	ATG8B check F	5' - GAAGGTTGCAAGCTATGGCGATTGAGGGAAACGGC - 3'
25	ATG8C check R	5' - GAATATAGCGATGATTTGCACCATAGATTCATAGG - 3'
26	ATG8D check F	5' - GTTTTACTGTAGATTGTTTGATAATCAAGGTAATC - 3'
27	ATG8F check F	5' - CTATTCCAGGAATTTCCAAATCAGCTTATCCATTC - 3'
28	EGFP check F	5' - TGCTATGCCTGAAGGTTATGTTCAAGAAAGAACTA- 3'
29	Pur4 check R	5' - TCAAGCACCAGGCTTTCTAG - 3'

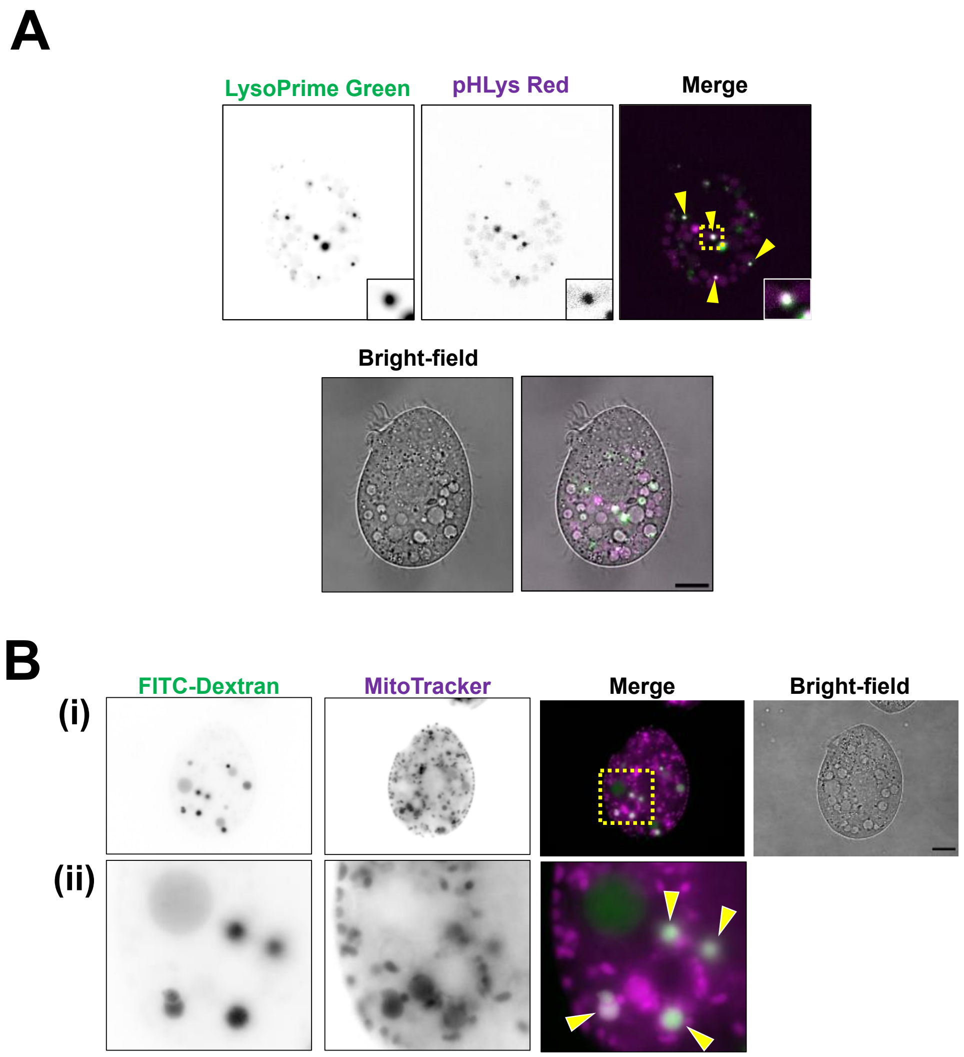

FIG S1 Acidity of lysosomes in *T. thermophila*. (A) Wild-type cells were starved for 2 h and stained with LysoPrime Green (green) and pHLys Red (magenta) for 30 min. Fluorescent images were captured using a Thunder imaging system. The bottom right panel shows a magnified view of the yellow dotted square. Scale bars: 10 µm. (B) Following 2 h of starvation, wild-type cells were fed 100 µg/mL FITC-dextran (green) and simultaneously stained with MitoTracker Red CMXRos (magenta) for 1 h. Prior to microscopic analysis, cells were washed with 10 mM Tris-HCl (pH7.5) to remove free FITC-dextran and MitoTracker Red CMXRos. Fluorescent images were obtained using an Olympus BX51 microscope. Scale bar: 10 µm (i). The bottom panel (ii) provides a magnified view of the yellow dotted square from the upper panel (i).

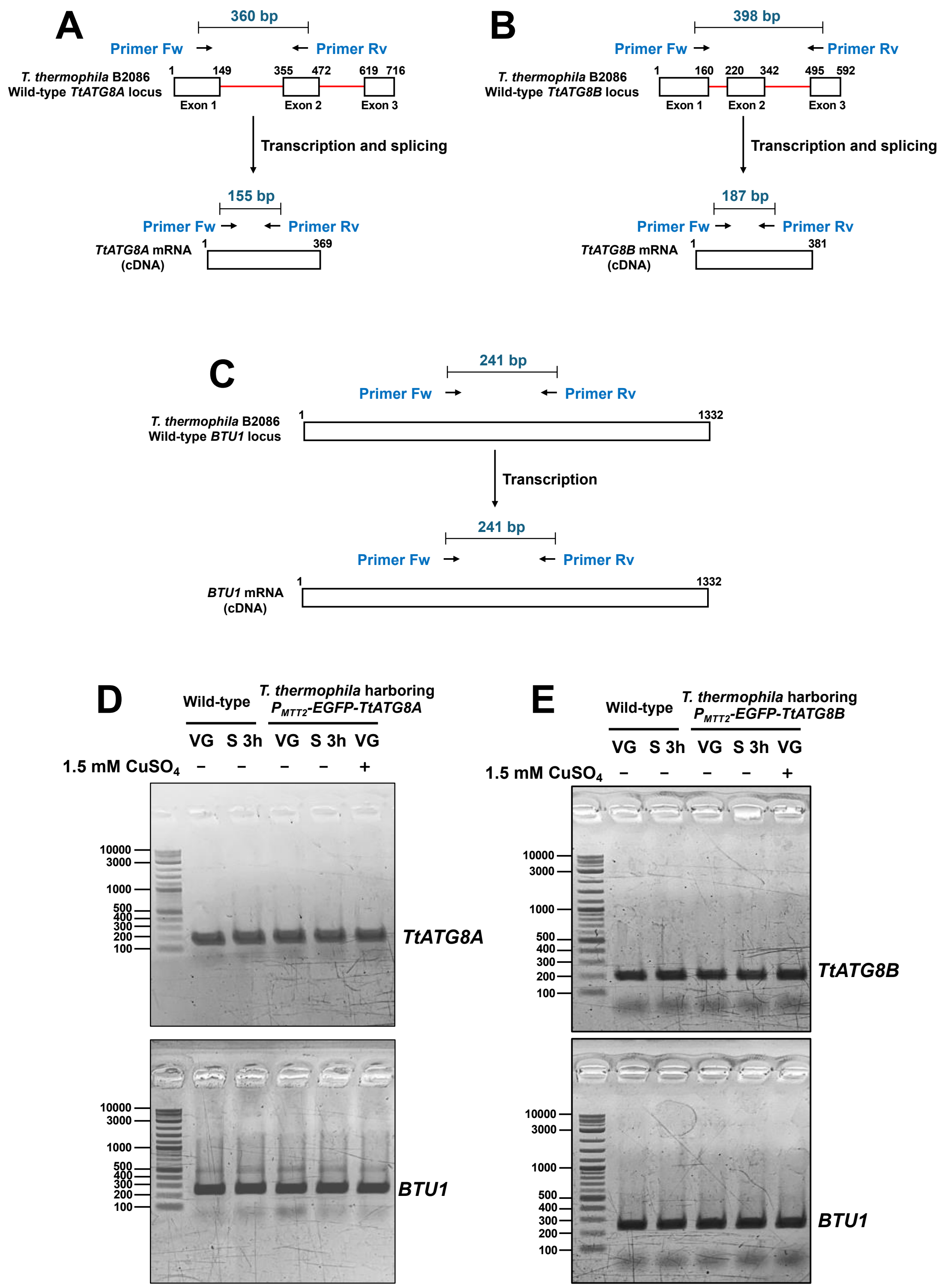

FIG S2 Schematic of RNA splicing and primer design for quantitative PCR analysis. (A–B) The primers targeting *TtATG8A* or *TtATG8B* were designed to span the intron. These primers generate a PCR product of 350–400 bp from genome DNA and 150–200 bp from RNA (cDNA),

respectively. (C) *BTU1* (*Beta Tubulin 1*) was employed as the internal standard since its expression levels were unaltered by nutrients starvation according to the TetraFGD database. The intron was not presented within *BTU1* genome locus. (D, E) Total RNA of vegetative growth cells (VG) and 3h starved cells (S 3h) were extracted and transcribed to cDNA. These cDNA were subjected to PCR analysis, and a single band of *TtATG8A*, or *TtATG8B* approximately 150–200 bp was detected.

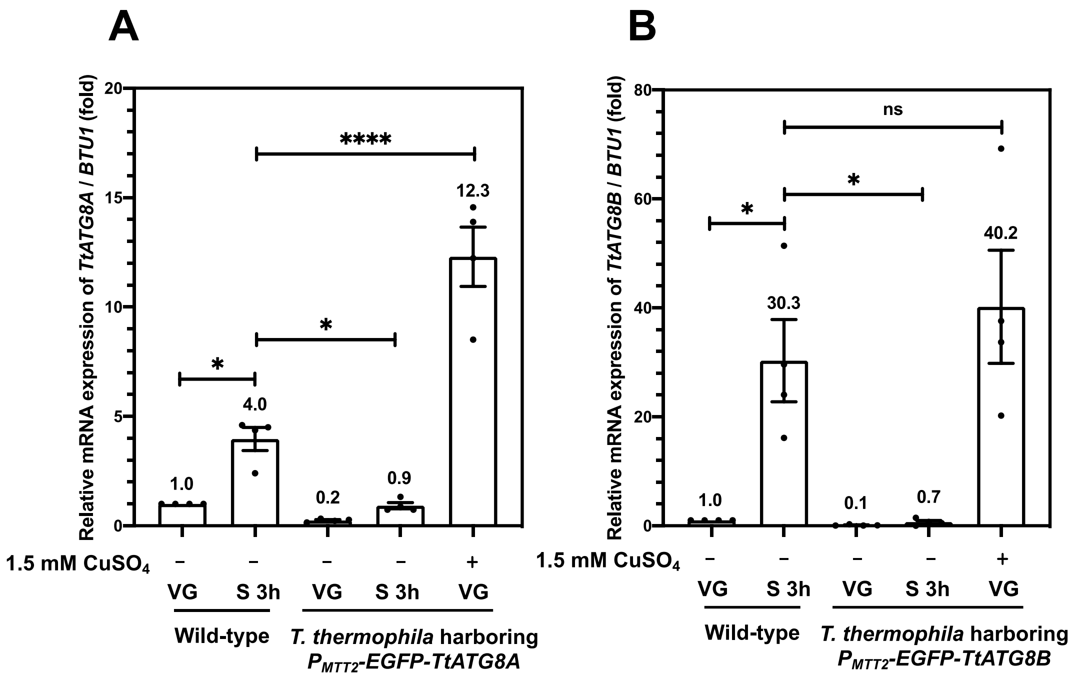

FIG S3 Quantitative PCR analysis of *TtATG8A* and *TtATG8B*. (A–B) Total RNA of vegetative growth cells (VG) and 3h starved cells (S 3h) were extracted and transcribed to cDNA. These cDNA were subjected to quantitative PCR analysis. mRNA levels of *BTU1* were monitored as the internal standard. Normalized mRNA levels of *TtATG8A* or *TtATG8A* of VG in wild-type cells was shown. Results are presented as means±s.e.m. from four independent experiments. One way ANOVA followed by Tukey's multiple comparison test was used, with *P < 0.05, ****P < 0.0001, *ns* indicating not significant.

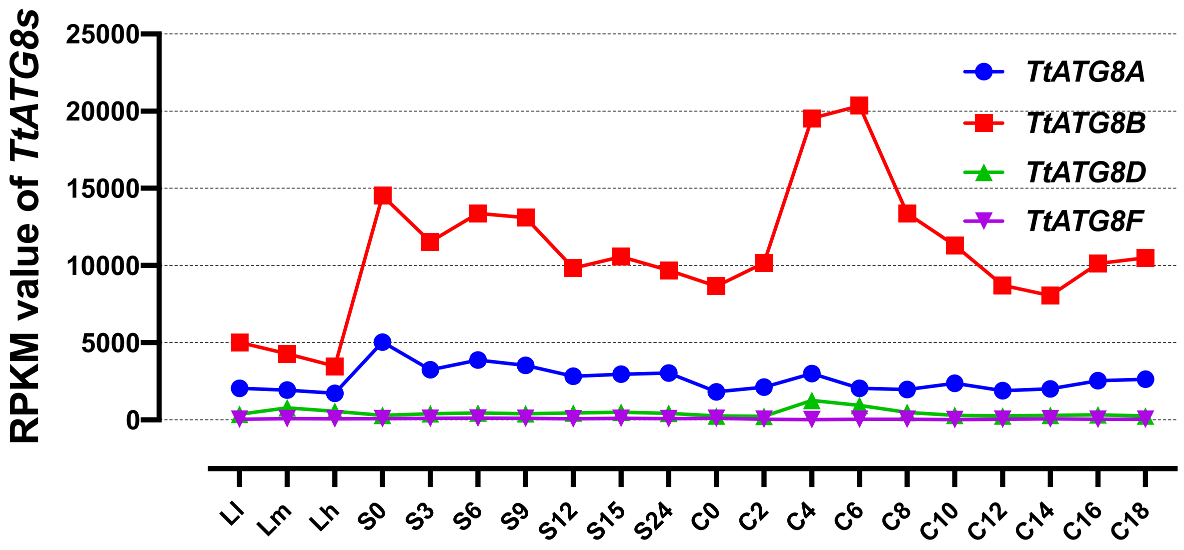

FIG S4 Expression pattern of *TtATG8s*. The transcription levels (RPKM values) of TtATG8s, as published in TetraFGD, are plotted. Ll, Lm, and Lh represent logarithmically growing cells at low, medium, and high densities, S0–S24 denotes the time points from 0–24 h after starvation of cells in Tris buffer, C0–C18 denotes the time points from 0–18 h after conjugation.

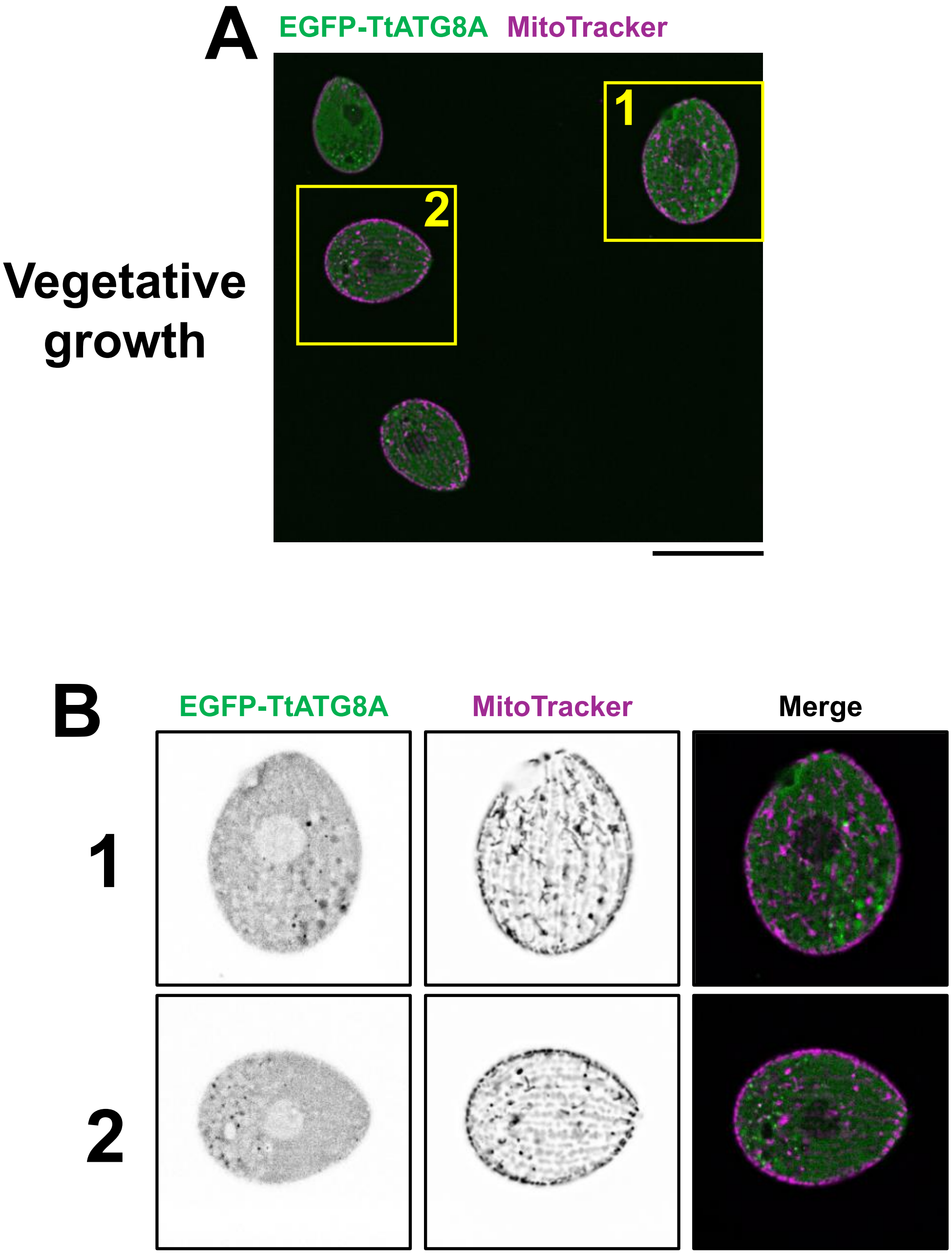

FIG S5 Localization pattern of EGFP-TtATG8A during vegetative growth. (A) Cells expressing the EGFP-TtATG8A constructs were cultured in SPP medium containing 1.5 mM $CuSO_4$ for 4 h. Log phase cells were stained with MitoTracker Red CMXRos for 1 h, and fluorescence images were obtained using an automated tile scan with Thunder imaging system. Scale bars: 50 μm. (B) Magnified view of the regions indicated by the yellow squares in (A). Green: EGFP-TtATG8A, Magenta: MitoTracker. The fluorescence images were focused on an

intermediate plane between the cortical and central regions of the cell due to focus limitations in wide-field tile scanning.

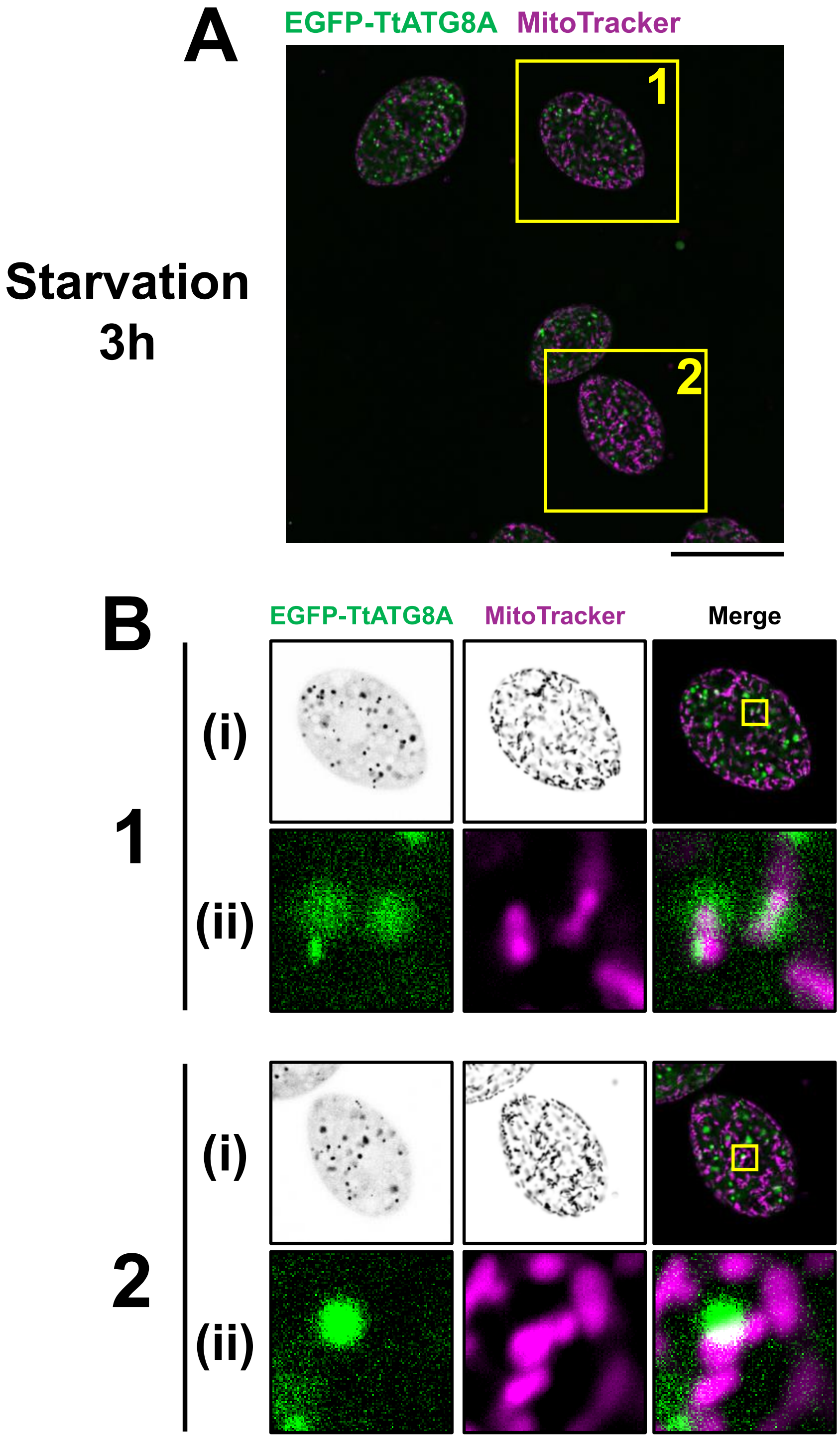

FIG S6 Localization pattern of EGFP-TtATG8A during starvation. Cells expressing the EGFP-TtATG8A constructs were cultured in SPP medium containing 1.5 mM $CuSO_4$ for 4 h. Three-hours starved cells were observed using an automated tile scan with Thunder imaging system. Scale bars: 50 µm. (B-(i)) Magnified view of the regions indicated by the yellow squares in

(A). (B-(ii)) Magnified view of the regions indicated by the yellow squares in (B-(i)).Green: EGFP-TtATG8A, Magenta: MitoTracker. The fluorescence images were focused on an intermediate plane between the cortical and central regions of the cell due to focus limitations in wide-field tile scanning.

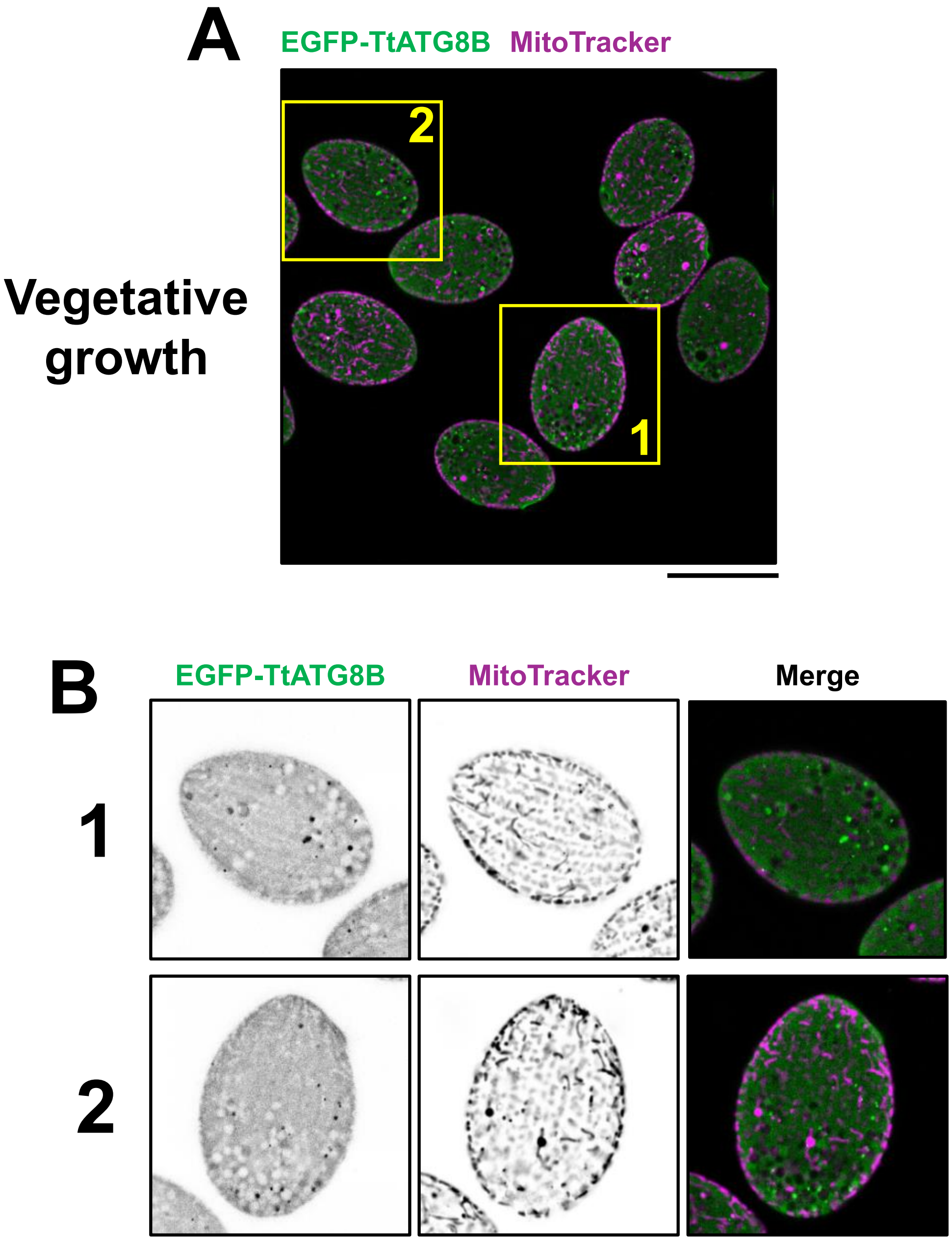

FIG S7 Localization pattern of EGFP-TtATG8B during vegetative growth. (A) Cells expressing the EGFP-TtATG8B constructs were cultured in SPP medium containing 1.5 mM $CuSO_4$ for 4 h. Log phase cells were stained with MitoTracker Red CMXRos for 1 h, and fluorescence images were obtained using an automated tile scan with Thunder imaging system. Scale bars: 50 μm. (B) Magnified view of the regions indicated by the yellow squares in (A). Green: EGFP-TtATG8B, Magenta: MitoTracker. The fluorescence images were focused on an

intermediate plane between the cortical and central regions of the cell due to focus limitations in wide-field tile scanning.

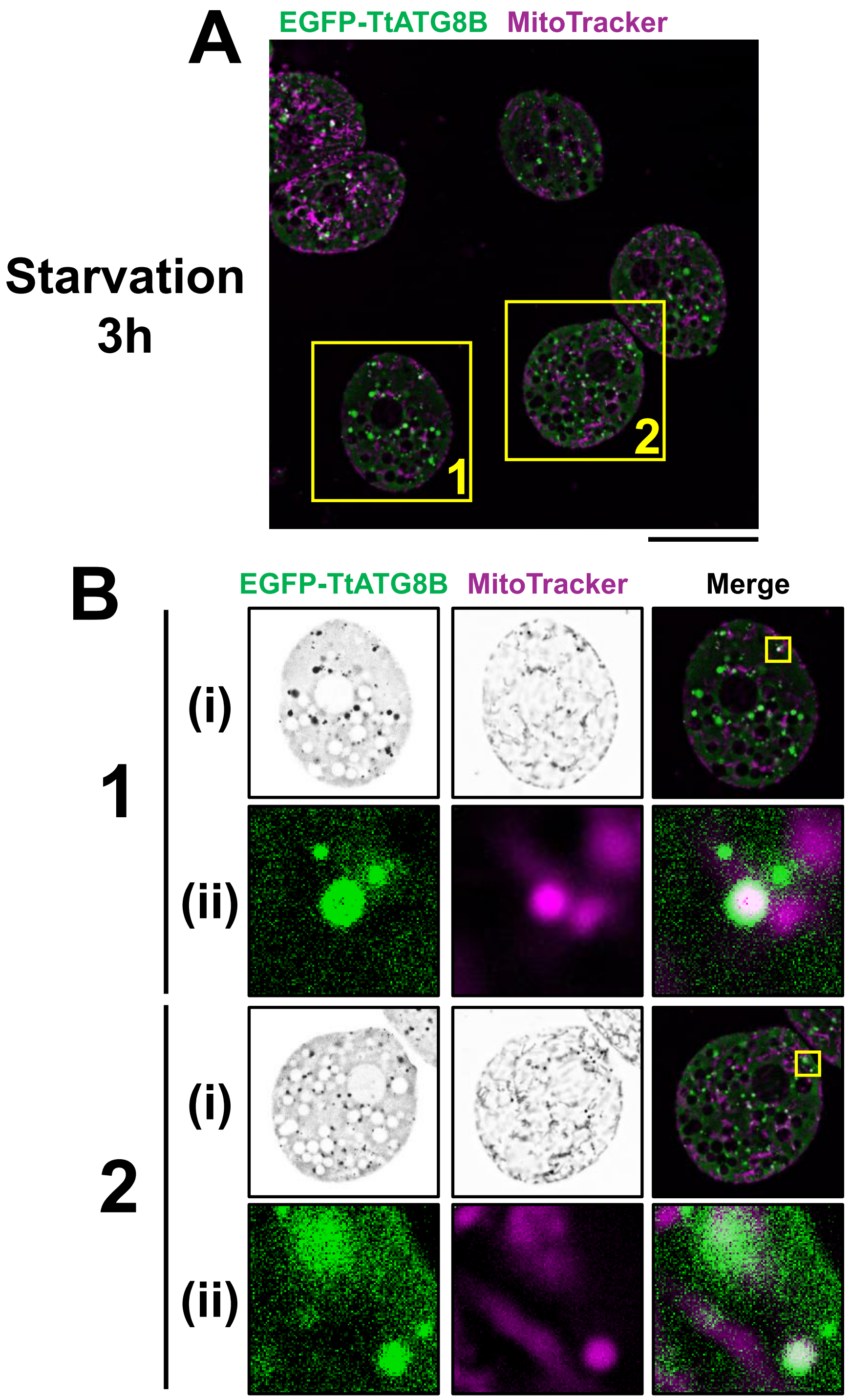

FIG S8 Localization pattern of EGFP-TtATG8B during starvation. Cells expressing the EGFP-TtATG8B constructs were cultured in SPP medium containing 1.5 mM $CuSO_4$ for 4 h. Three-hours starved cells were observed using an automated tile scan with Thunder imaging system. Scale bars: 50 µm. (B) Magnified view of the region indicated by the yellow square in (A). (B-

(i)) Magnified view of the regions indicated by the yellow square in (A). (B-(ii)) Magnified view of the regions indicated by the yellow squares in (B-(i)). Green: EGFP-TtATG8A, Magenta: MitoTracker. Green: EGFP-TtATG8B, Magenta: MitoTracker. The fluorescence images were focused on an intermediate plane between the cortical and central regions of the cell due to focus limitations in wide-field tile scanning.

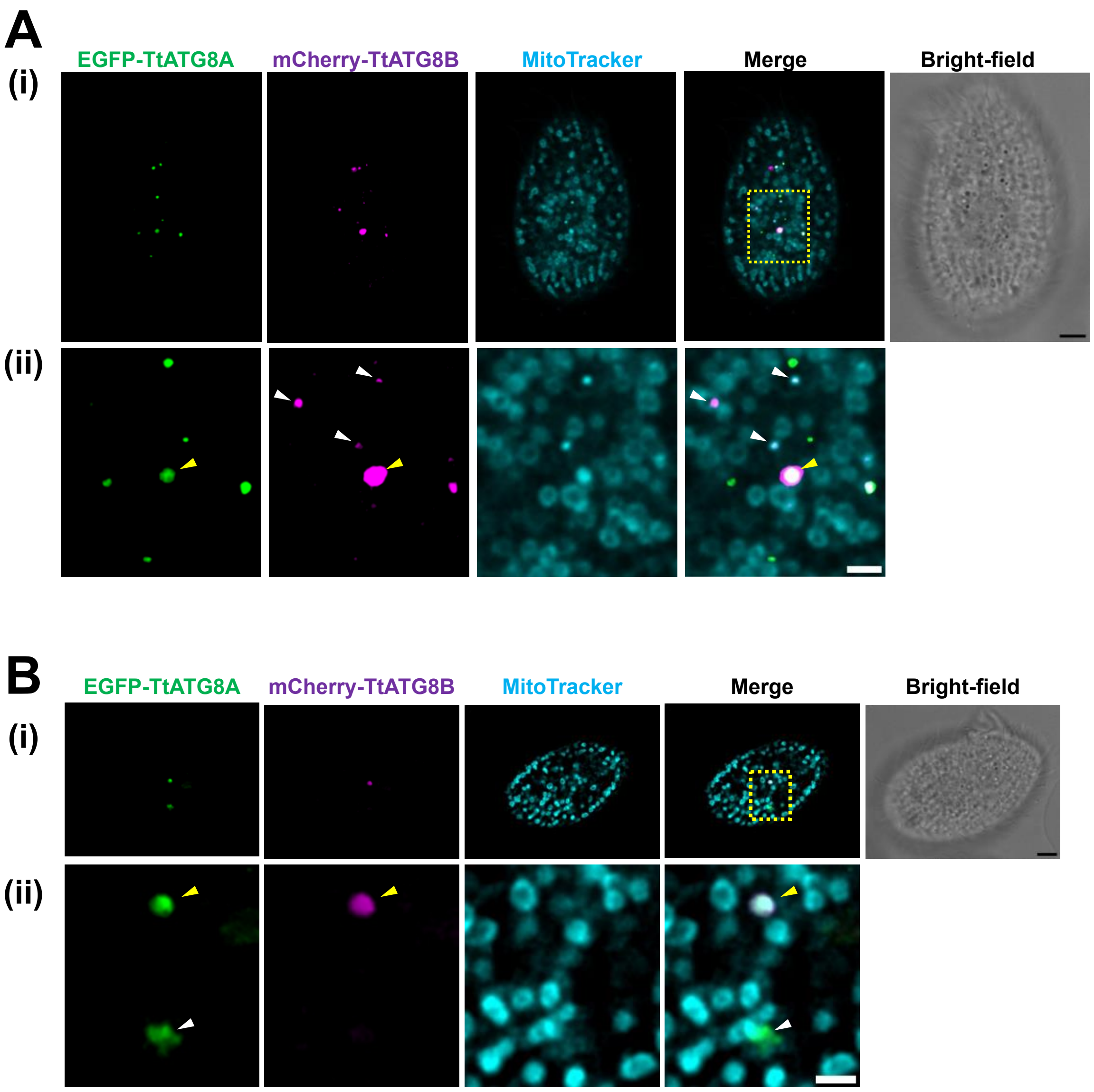

FIG S9 Localization of *TtATG8A* and *TtATG8B*. (A, B) *T. thermophila* strains expressing *EGFP-TtATG8A* and *mCherry-TtATG8B* strains were cultured in SPP with 1.5 mM $CuSO_4$ and starved for 2 h. The cells were then stained with 200 nM Mitotracker DeepRed for 1 h, fixed with 2% paraformaldehyde, and fluorescence images were captured using a TCS SP8 confocal microscope. Green: EGFP-TtATG8A, Magenta: mCherry-TtATG8B, Cyan: MitoTracker. The bottom panel (ii) shows a magnified view of the yellow dotted square from the upper panel (i). Scale bars: 5 μm (i) and 2 μm (ii).

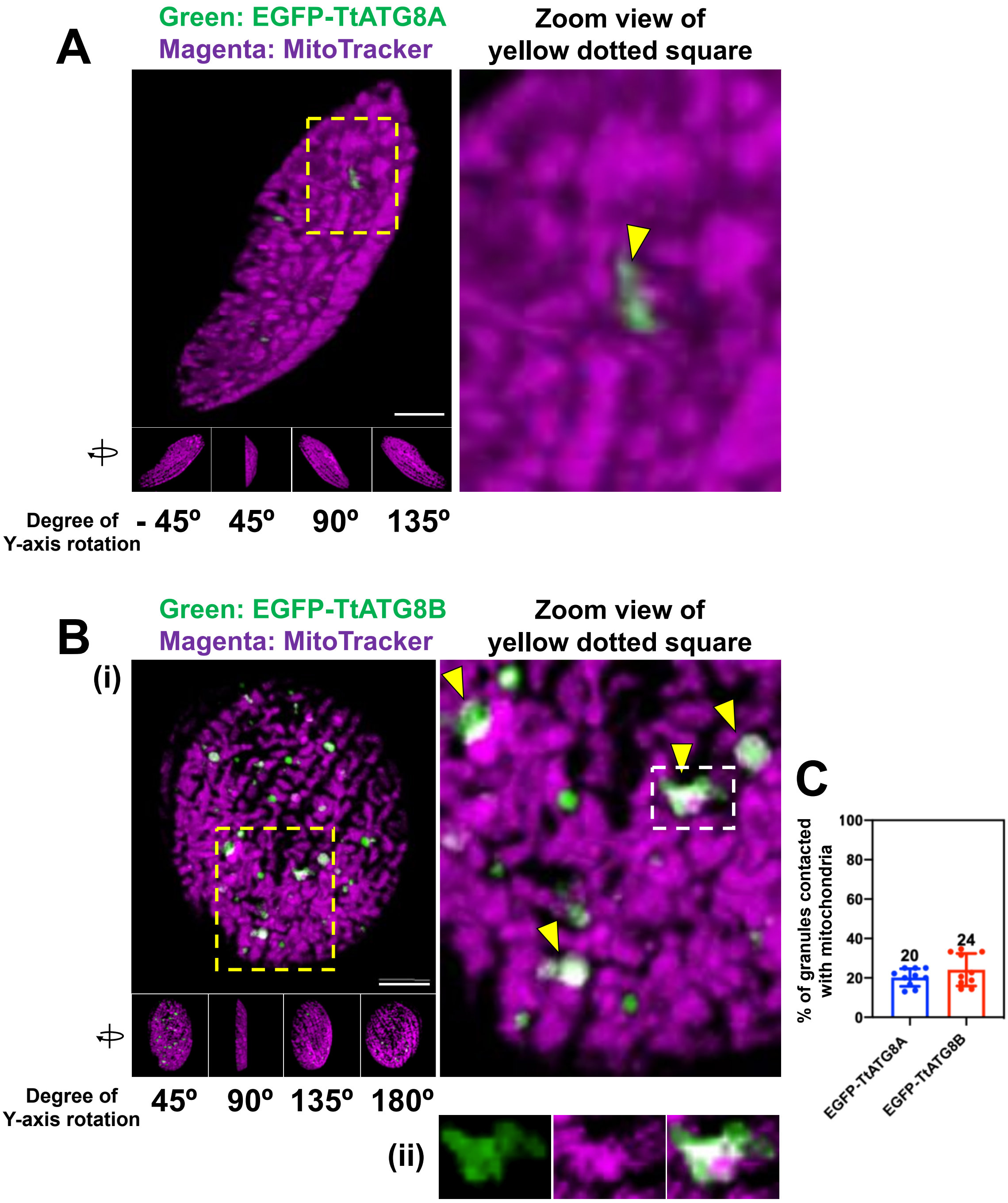

FIG S10 Interaction of TtATG8A and TtATG8B with mitochondria. (A, B) *T. thermophila* strains expressing *EGFP-TtATG8A* or *EGFP-TtATG8B* were cultured in SPP with 1.5 mM $CuSO_4$. Cells were starved for 2.5 hours, stained with 200 nM MitoTracker Red CMXRos for 30 min, and fixed with 2% paraformaldehyde and 0.5% glutaraldehyde. Fluorescent images were obtained using a confocal microscope (FV3000). Green; EGFP-TtATG8A, Magenta; MitoTracker. The 3D images were generated using Fiji software. Scale bar: 5 µm. The bottom

panel (ii) displays a magnified view of the white dotted square from the upper panel (i). (C) The graph shows the percentage of granules where each protein is in contact or very close to mitochondria. The granular EGFP signal overlapping with MitoTracker signal was assessed by the presence of white regions (merged color of green and magenta), as indicated by yellow arrowheads in (B). Results are expressed as the mean ± s.e.m (*n* = 10 cells).

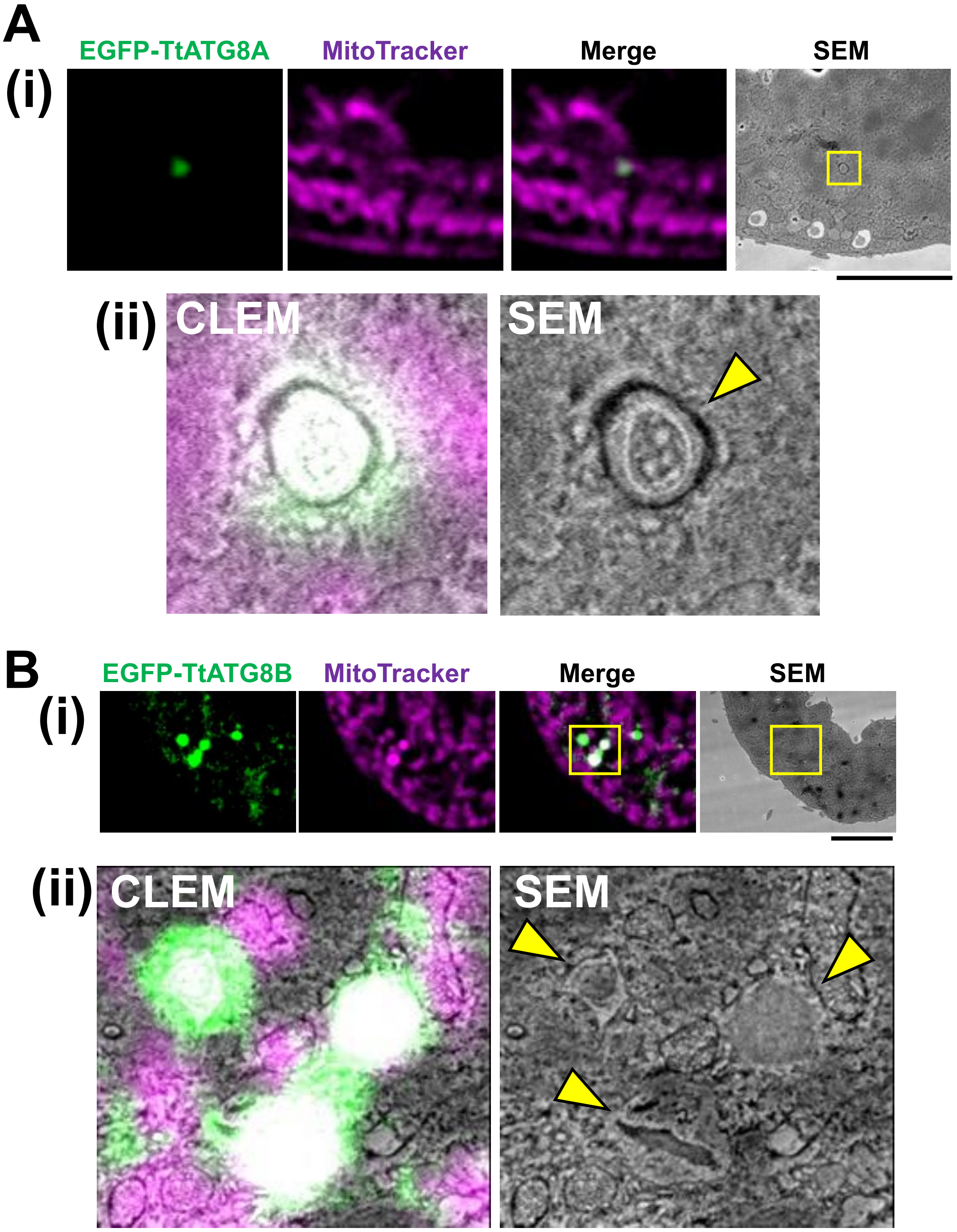

FIG S11 CLEM analysis of TtATG8A and TtATG8B. (A, B) Fluorescence, SEM, and CLEM images of *T. thermophila* expressing EGFP-TtATG8A (A) and EGFP-TtATG8B (B) after 3 h of starvation. The bottom panel (ii) shows a digitally magnified view of the yellow square from the upper panel (i). Green: EGFP-TtATG8A or B, Magenta: MitoTracker. Scale bars: 5 µm.

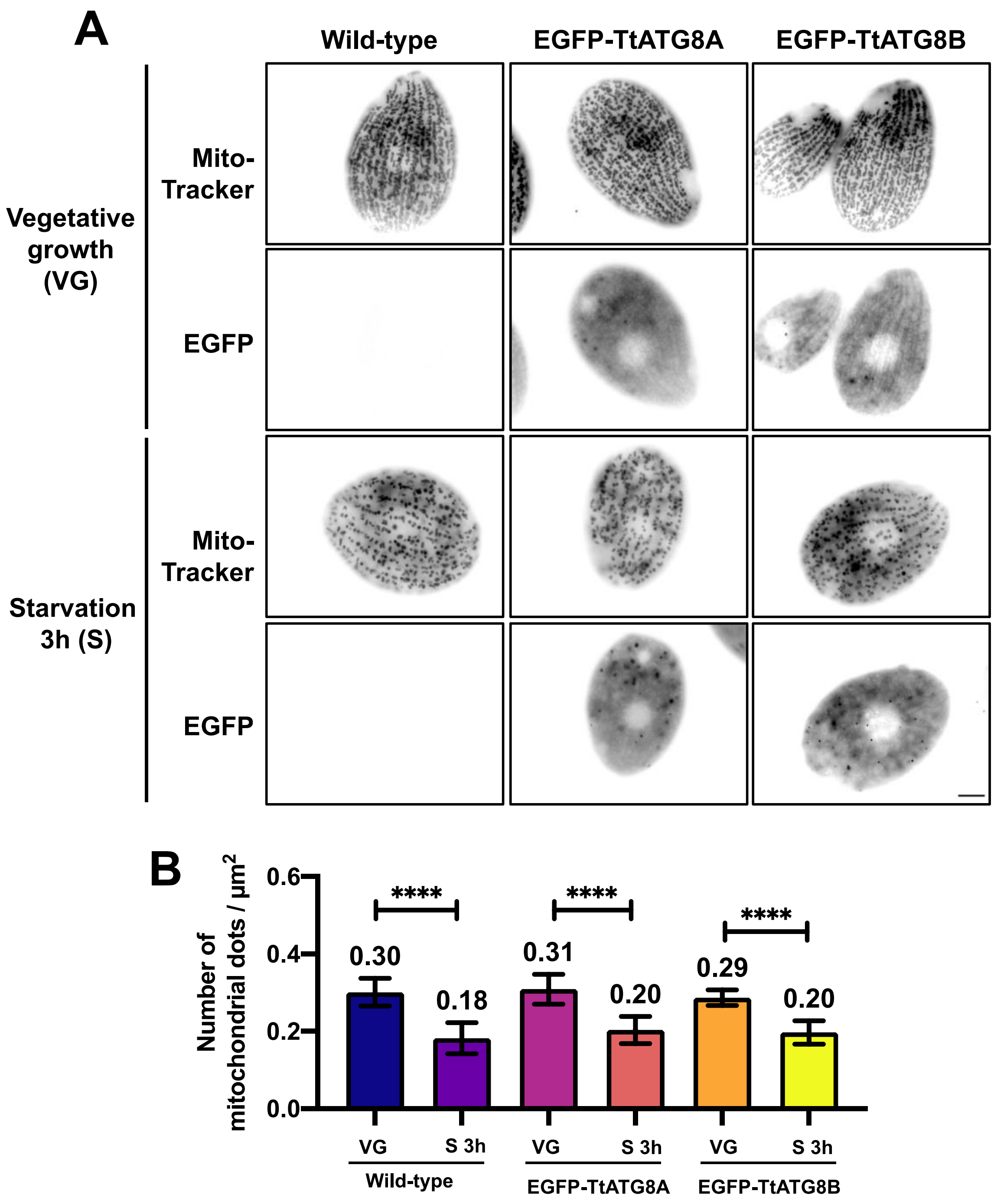

FIG S12 Mitochondrial degradation in cells expressing EGFP-*TtATG8A* or EGFP-*TtATG8B* after starvation. (A, B) *T. thermophila* wild-type cells and cells harboring *EGFP-TtATG8* were cultured overnight in SPP medium containing 1.5 mM $CuSO_4$. Vegetative growth cells (VG) and starved cells (S) were stained with 200 nM MitoTracker Red CMXRos. Fluorescent images, focusing on the cell surface, were captured using an Olympus BX51 microscope. The number of mitochondrial dots and the cell area (μm^2) were quantified using Image J software. Results

are expressed as the mean ± s.e.m (*n* = 20 cells). Statistical significance was determined using one way ANOVA followed by Tukey's multiple comparison test: ****$P < 0.0001$, *ns* indicates not significant. Scale bars: 10 μm.

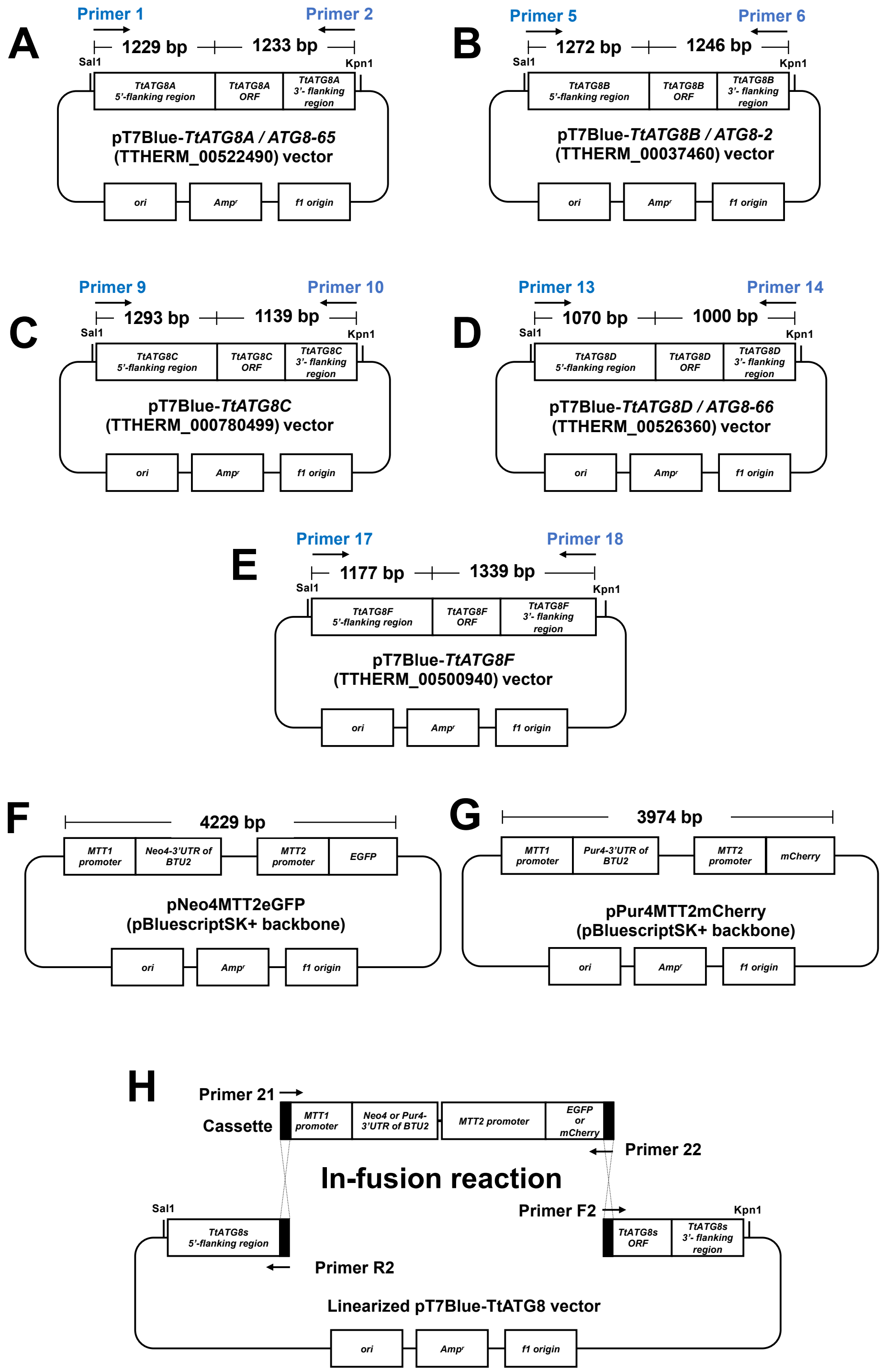

FIG S13 Vector map 1. (A–E) Approximately 2.5 kb regions, including the *TtATG8s* open reading frame (ORF) along with about 1 kb of upstream and downstream regions, were amplified using PrimeSTAR Max DNA polymerase (Takara) with genomic DNA extracted from *T. thermophila* wild-type cells as the template. The amplified genes were cloned into the pT7Blue vector (Merck Millipore). (F and G) To construct the N-terminal tagging vectors, the *MTT2* promoter (P_{MTT2}) (see Supporting references; 1) and a fluorescent protein gene were

inserted at the 3'end of the Neo4 cassette in the pNeo4 vector (see Supporting references; 2) through gene manipulation techniques, including the addition of restriction sites by site-directed mutagenesis and gene ligation. The resultant vectors, named pNeo4MTT2eGFP and pPur4MTT2mCherry, respectively, were used for N-terminal fluorescent protein-tagging. (H) To tag the N-terminus of TtATG8s with EGFP or mCherry, cassettes comprising P_{MTT1}*-Neo4-*P_{MTT2}*-EGFP* or P_{MTT1}*-Neo4-*P_{MTT2}*-mCherry* were amplified by PCR from the vectors shown in F and G, respectively. Meanwhile, a linearized pT7Blue -*TtATG8s* vector was prepared by inverse PCR. The cassette was then fused with the linearized pT7Blue-*TtATG8s* vector using the In-fusion HD Cloning kit (Takara).

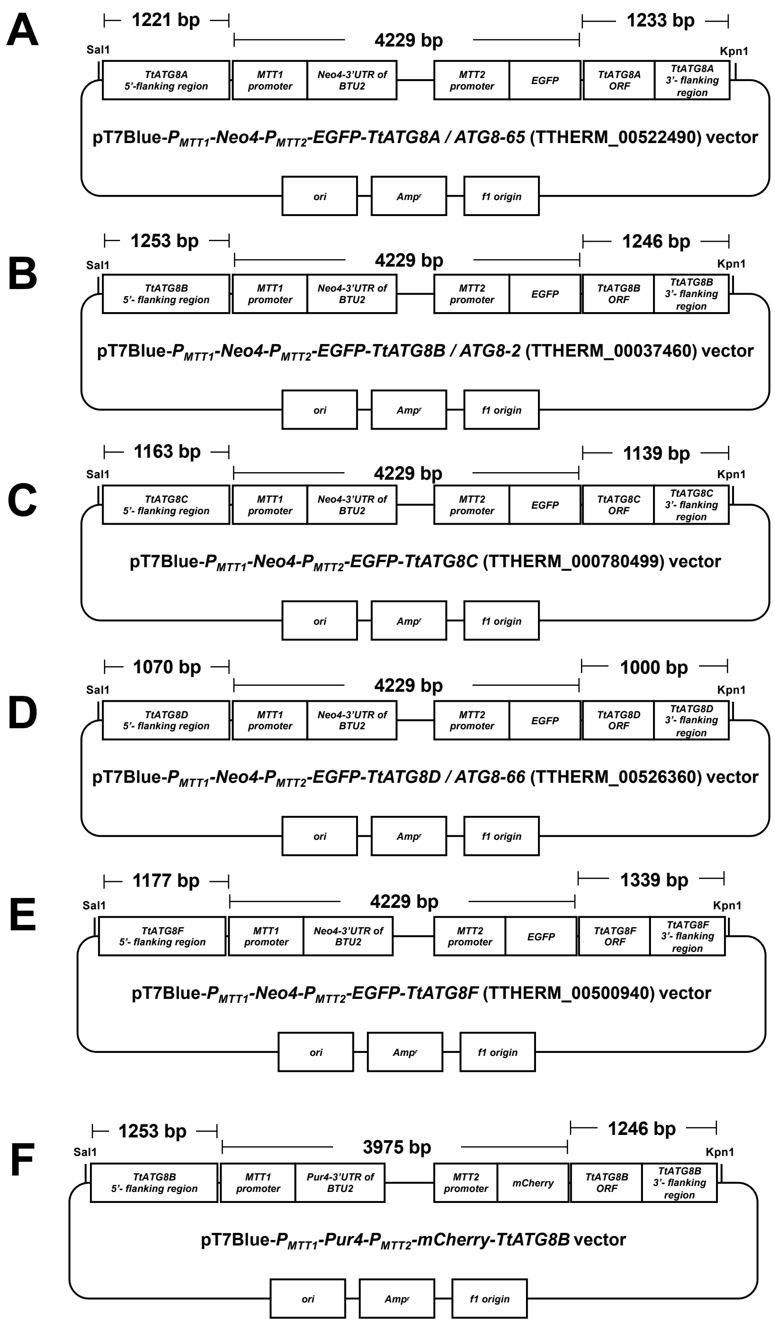

FIG S14 Vector map 2 (plasmids for transformation). (A–F) Maps of the vectors generated using the method depicted in Fig. S13 are presented.

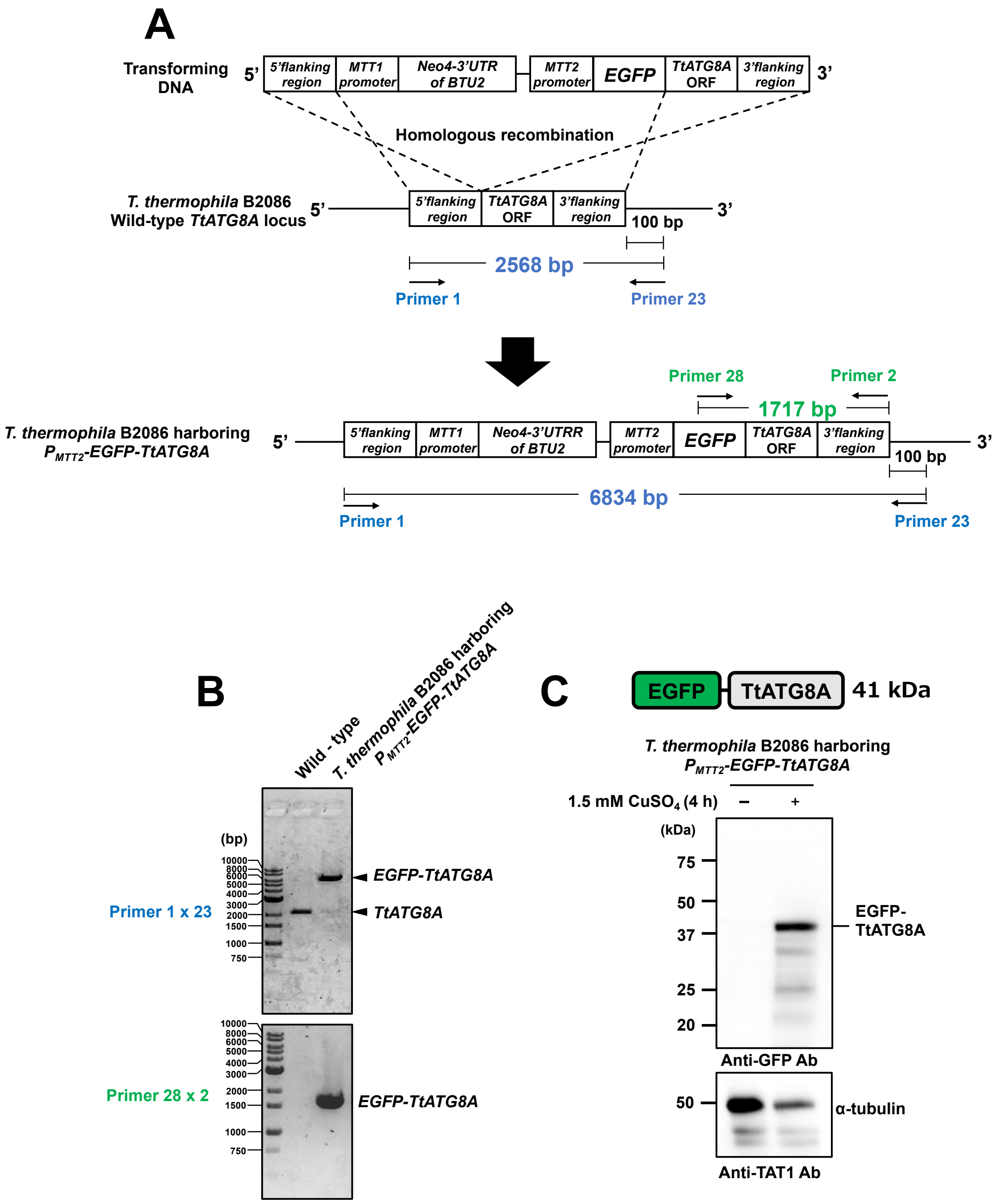

FIG S15 A *T. thermophila* strain harboring *EGFP-TtATG8A*. (A) Overview of the transformation process. (B) Confirmation of genetic recombination. Genomic DNA of *T. thermophila* harboring *EGFP-TtATG8A* was analyzed by PCR using the indicated primer pairs. (C) Transformed cells were cultured in SPP medium, with or without 1.5 mM $CuSO_4$, for 4 h at 30°C. Cell lysates were separated by SDS-PAGE (without urea) and subjected to immunoblotting using anti-GFP and anti-TAT1 antibodies. α-tubulin was used as a protein loading control.

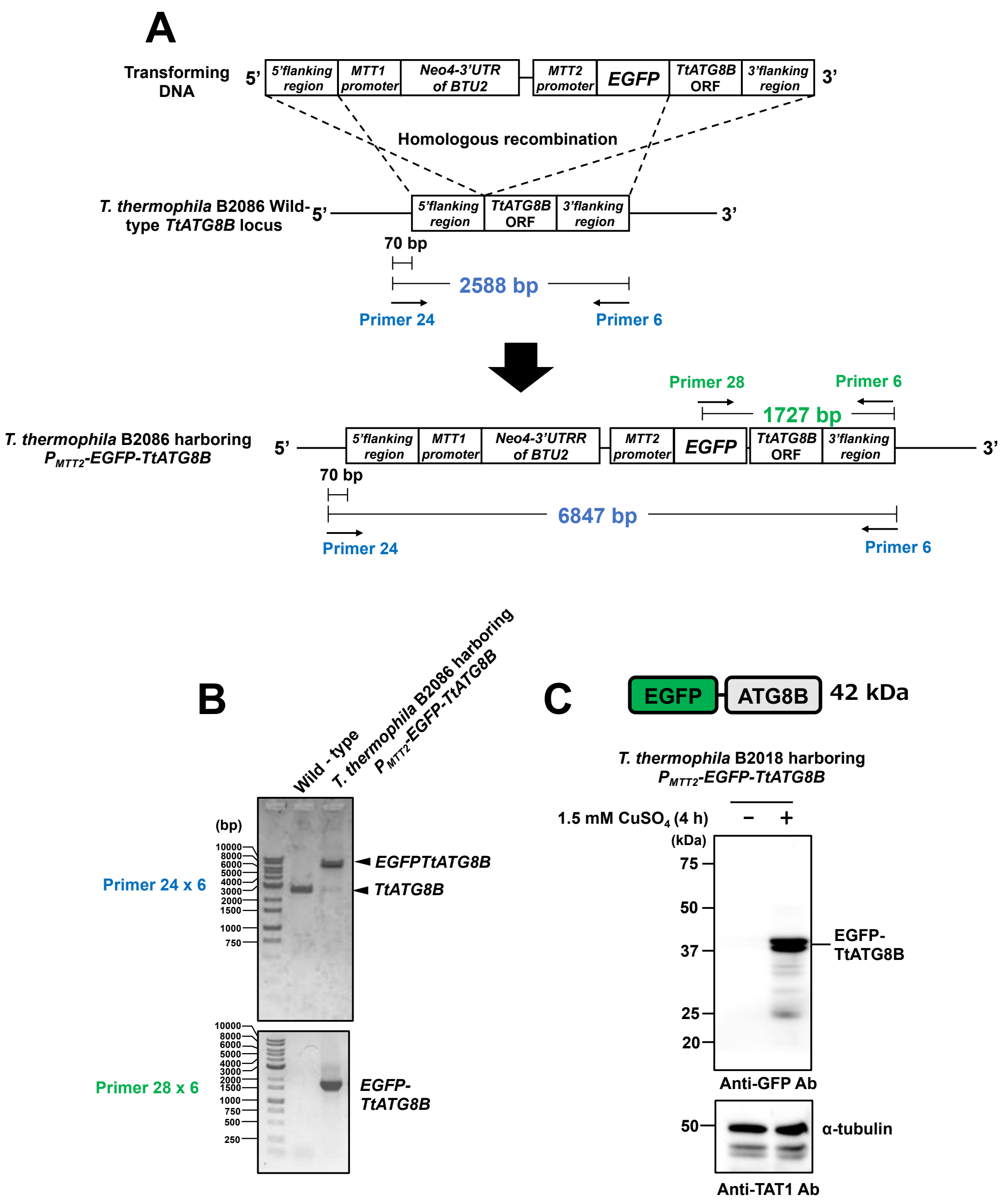

FIG S16 A *T. thermophila* strain harboring *EGFP-TtATG8B*. (A) Overview of the transformation process. (B) Confirmation of genetic recombination. Genomic DNA from *T. thermophila* harboring *EGFP-TtATG8B* was analyzed by PCR using the indicated primer pairs. (C) Transformed cells were cultured in SPP medium, with or without 1.5 mM $CuSO_4$, for 4 h at 30°C. Cell lysates were separated by SDS-PAGE (without urea) and subjected to immunoblotting using anti-GFP and anti-TAT1 antibodies. α-tubulin was used as a protein loading control.

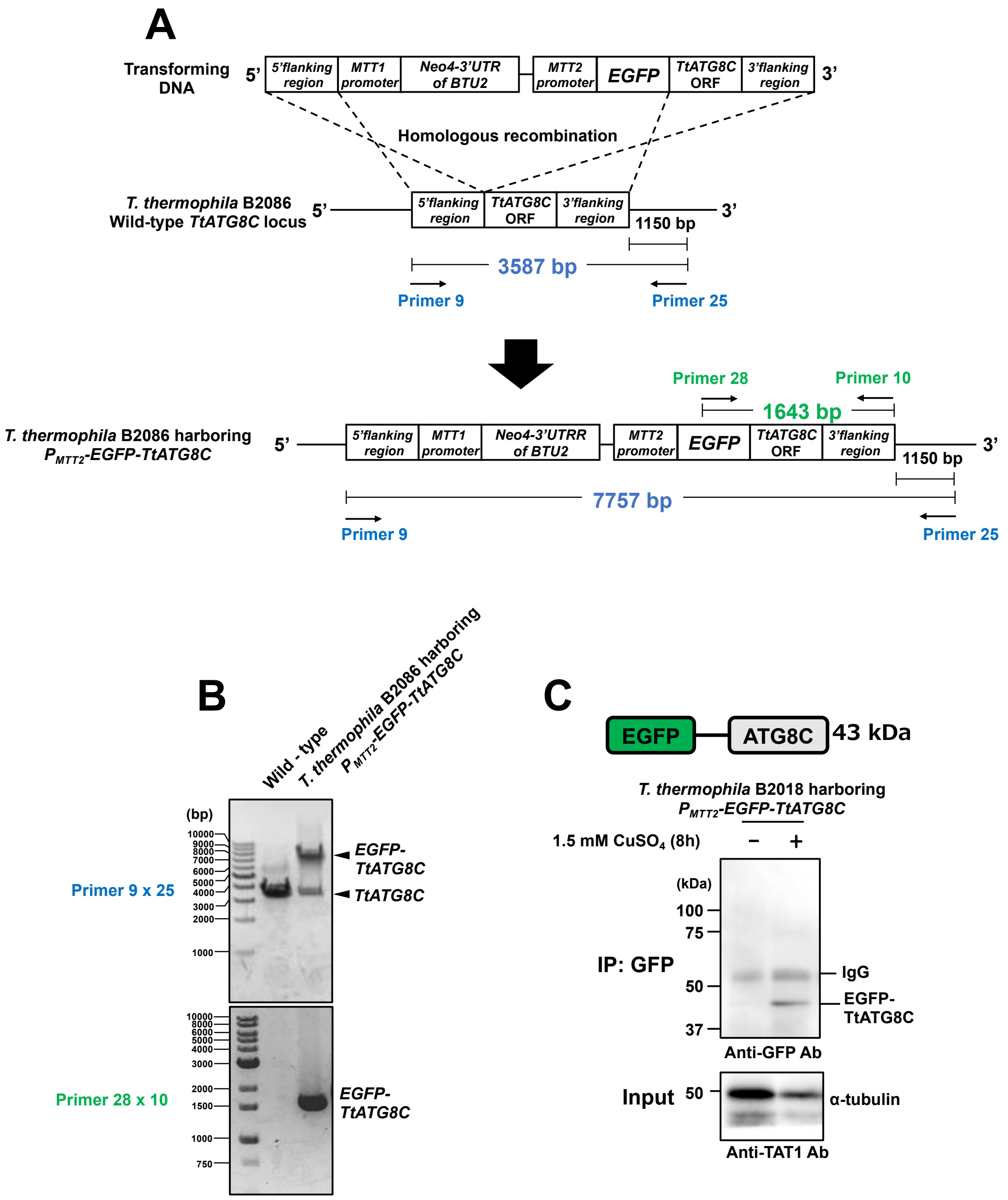

FIG S17 A *T. thermophila* strain harboring *EGFP-TtATG8C*. (A) Overview of the transformation process. (B) Confirmation of genetic recombination: Genomic DNA from *T. thermophila* harboring *EGFP-TtATG8C* was analyzed by PCR using the indicated primer pairs. (C) Transformed cells were cultured in SPP medium, with or without 1.5 mM $CuSO_4$, for 8 h at 30°C. Cell lysates were immunoprecipitated with an anti-GFP antibody (represented as IP: GFP). Both the immunoprecipitants and cell lysates before immunoprecipitation (denoted as Input) were separated by SDS-PAGE (without urea) and subjected to immunoblotting using anti-GFP and anti-TAT1 antibodies, respectively. α-tubulin was used as a protein loading control.

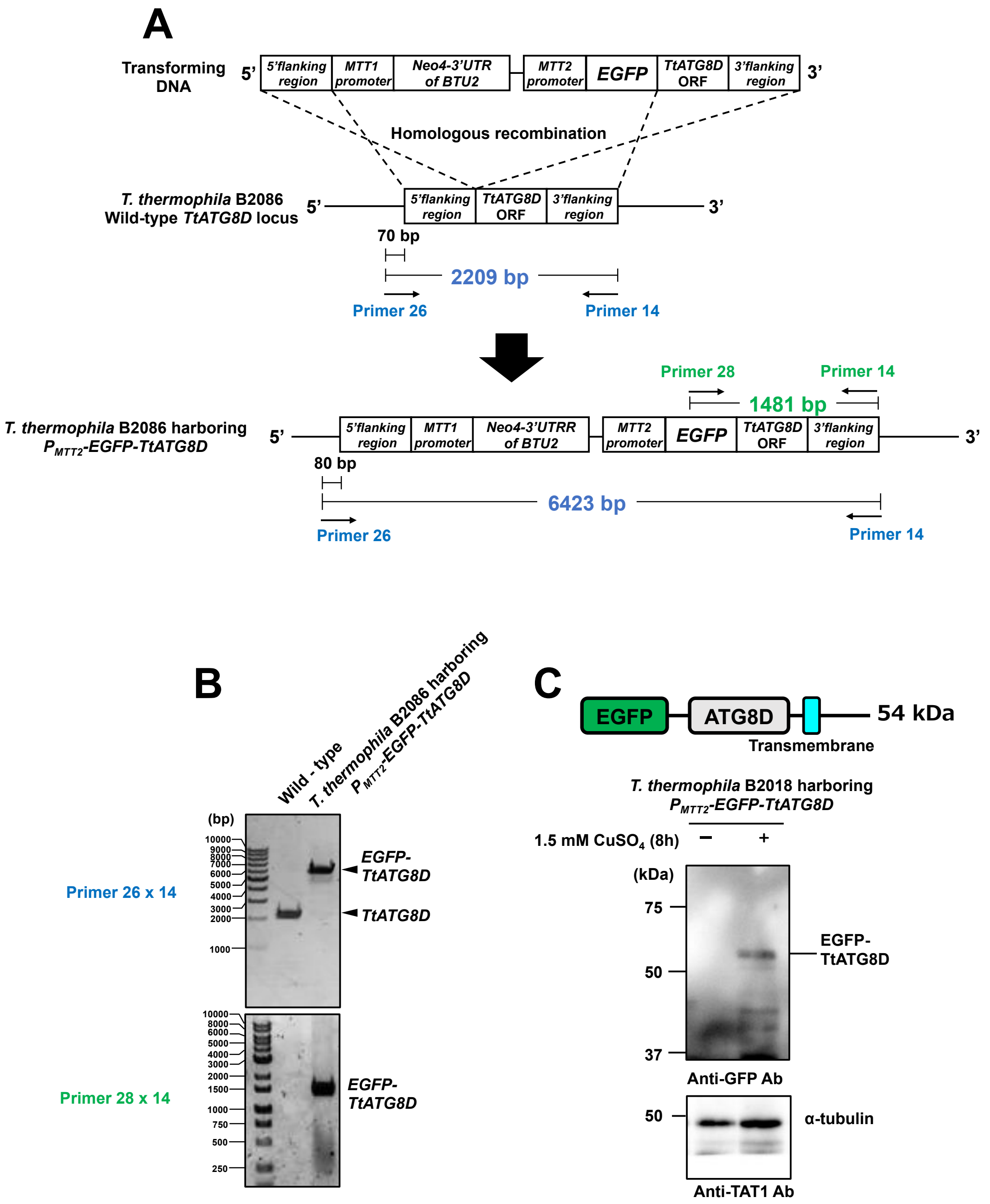

FIG S18 A *T. thermophila* strain harboring *EGFP-TtATG8D*. (A) Overview of the transformation process. (B) Confirmation of genetic recombination: Genomic DNA from *T. thermophila* harboring *EGFP-TtATG8D* was analyzed by PCR using the indicated primer pairs. (C) Transformed cells were cultured in SPP medium, with or without 1.5 mM $CuSO_4$, for 8 h at 30°C. Cell lysates were separated by SDS-PAGE (without urea), and subjected to immunoblotting using anti-GFP and anti-TAT1 antibodies. α-tubulin was used as a protein loading control.

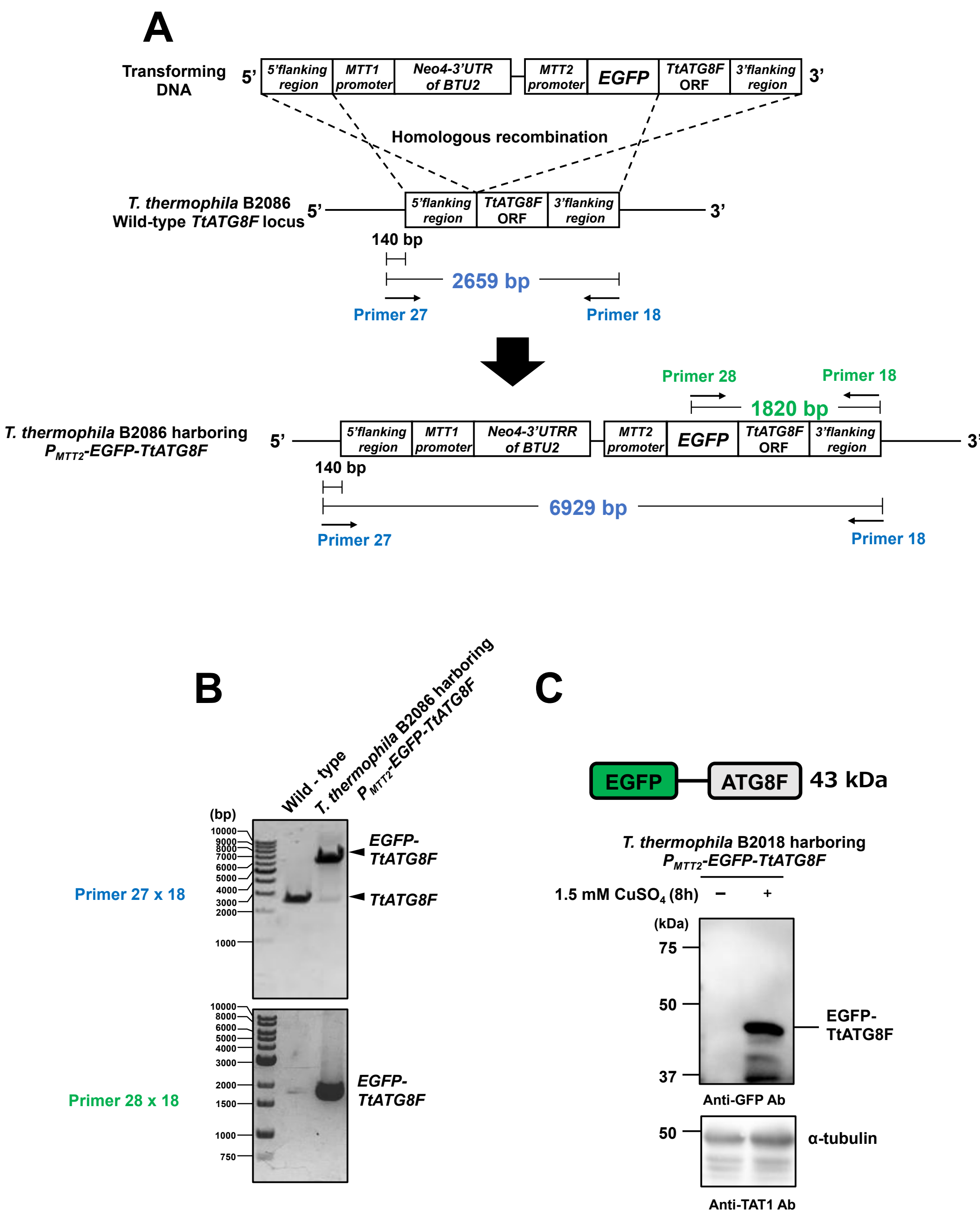

FIG S19 A *T. thermophila* strain harboring *EGFP-TtATG8F*. (A) Overview of the transformation process. (B) Confirmation of genetic recombination: Genomic DNA from *T. thermophila* harboring *EGFP-TtATG8F* was analyzed by PCR using the specified primer pairs. (C) Transformed cells were cultured in SPP medium, with or without 1.5 mM $CuSO_4$, for 8 h at 30°C. Cell lysates were separated by SDS-PAGE (without urea) and subjected to immunoblotting using anti-GFP and anti-TAT1 antibodies. α-tubulin was used as a protein loading control.

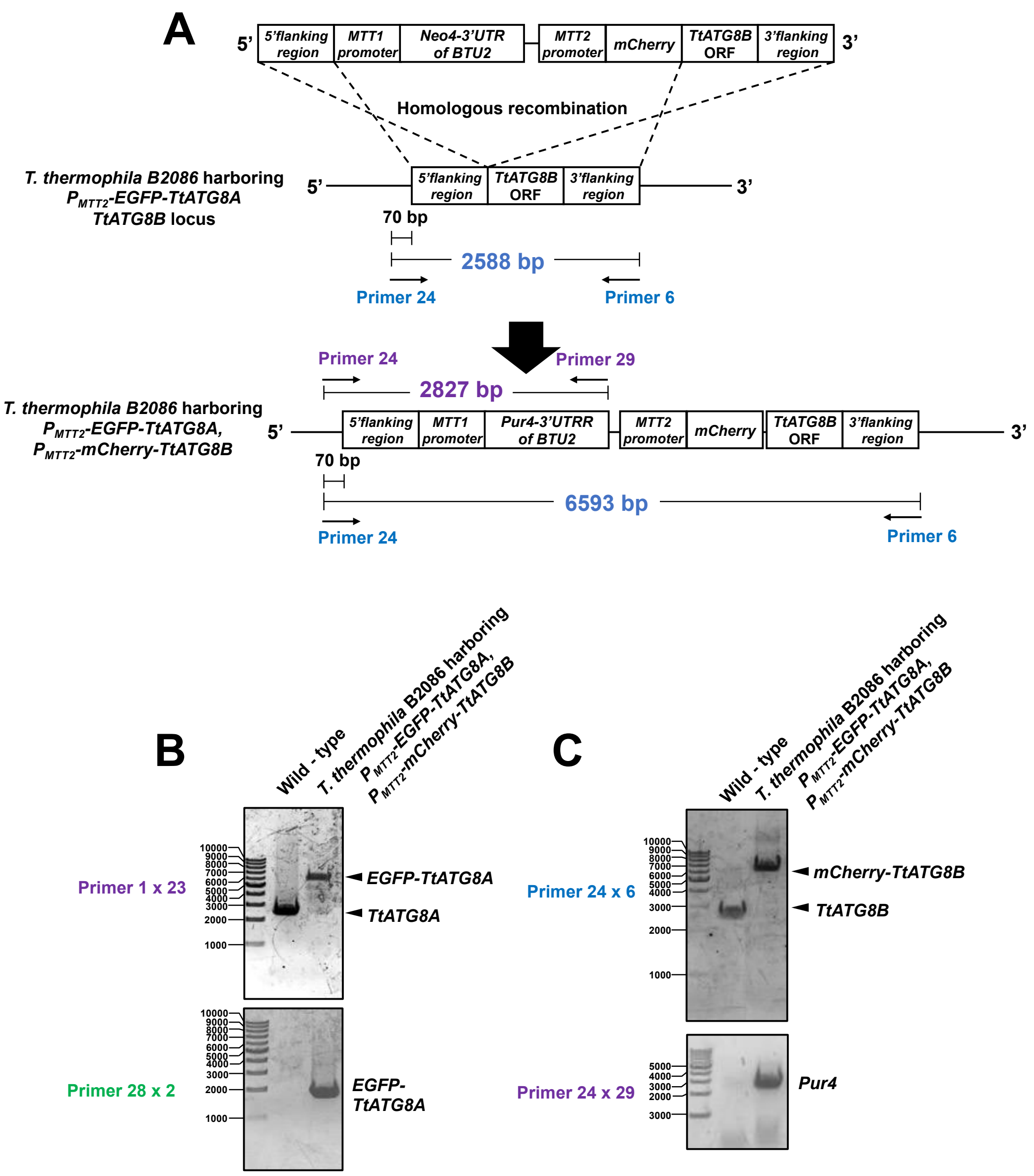

FIG S20 A *T. thermophila* strain harboring *EGFP-TtATG8A* and *mCherry-TtATG8B*. (A) Overview of transformation. (B, C) Confirmation of genetic recombination. Genomic DNA of *T. thermophila* harboring *EGFP-TtATG8A* and *mCherry-TtATG8B* was subjected to PCR, using the indicated primer pairs.

Supporting References

1. **Boldrin F, Santovito G, Formigari A, Bisharyan Y, Cassidy-Hanley D, Clark TG, Piccinni E.** 2008. MTT2, a copper-inducible metallothionein gene from *Tetrahymena thermophila*. *Comparative Biochemistry and Physiology Part C: Toxicology & Pharmacology* 147:232–240. 10.1016/j.cbpc.2007.10.002.

2. **Mochizuki K.** 2008. High efficiency transformation of *Tetrahymena* using a codon-optimized neomycin resistance gene. *Gene* 425:79–83. 10.1016/j.gene.2008.08.007.
